# Insight into aphid mediated *Potato Virus Y* transmission: A molecular to bioinformatics prospective

**DOI:** 10.3389/fmicb.2022.1001454

**Published:** 2022-11-24

**Authors:** Tanmaya Kumar Bhoi, Ipsita Samal, Prasanta Kumar Majhi, J. Komal, Deepak Kumar Mahanta, Asit Kumar Pradhan, Varun Saini, M. Nikhil Raj, Mohammad Abbas Ahmad, Partha Pratim Behera, Mangali Ashwini

**Affiliations:** ^1^Forest Protection Division, ICFRE-Arid Forest Research Institute (AFRI), Jodhpur, Rajasthan, India; ^2^Department of Entomology, Sri Sri University, Cuttack, Odisha, India; ^3^Department of Plant Breeding and Genetics, Odisha University of Agriculture and Technology, Bhubaneswar, Odisha, India; ^4^Department of Entomology, Navsari Agricultural University, Navsari, Gujarat, India; ^5^Department of Entomology, Dr. Rajendra Prasad Central Agricultural University, Samastipur, India; ^6^Social Science Division, ICAR-National Rice Research Institute (NRRI), Cuttack, Odisha, India; ^7^Division of Entomology, ICAR-Indian Agricultural Research Institute (IARI), New Delhi, India; ^8^Assam Agricultural University, Jorhat, Assam, India

**Keywords:** potato, *Potato Virus Y* (PVY), vector, molecular, bioinformatics, transcriptome, genome, aphid

## Abstract

Potato, the world's most popular crop is reported to provide a food source for nearly a billion people. It is prone to a number of biotic stressors that affect yield and quality, out of which *Potato Virus Y* (PVY) occupies the top position. PVY can be transmitted mechanically and by sap-feeding aphid vectors. The application of insecticide causes an increase in the resistant vector population along with detrimental effects on the environment; genetic resistance and vector-virus control are the two core components for controlling the deadly PVY. Using transcriptomic tools together with differential gene expression and gene discovery, several loci and genes associated with PVY resistance have been widely identified. To combat this virus we must increase our understanding on the molecular response of the PVY-potato plant-aphid interaction and knowledge of genome organization, as well as the function of PVY encoded proteins, genetic diversity, the molecular aspects of PVY transmission by aphids, and transcriptome profiling of PVY infected potato cultivars. Techniques such as molecular and bioinformatics tools can identify and monitor virus transmission. Several studies have been conducted to understand the molecular basis of PVY resistance/susceptibility interactions and their impact on PVY epidemiology by studying the interrelationship between the virus, its vector, and the host plant. This review presents current knowledge of PVY transmission, epidemiology, genome organization, molecular to bioinformatics responses, and its effective management.

## Introduction

Potato (*Solanum tuberosum* L.) is a fundamental source of nutrient-providing food crops across the world. It occupies the third position globally in terms of consumption after rice and wheat (Soare and Chiurciu, 2021). Like other crops, potato production and productivity are also affected by diverse biotic and abiotic stresses that lead to yield losses (Bond, 2014). The major abiotic stressors that affect potato crops are drought, heat, nutrient deficiency, salinity, and cold/frost stress. Some biotic stressors cause diseases like late blight and bacterial wilt; among insect pests, sucking pests include aphids, thrips, whiteflies, mites, hoppers, borers like potato tuber moths, and potato cyst nematodes (Singh et al., 2020) and are considered to be utmost important (Handayani et al., 2019). Generally, the potato has been reported to be susceptible to a high number of pathogens and pests including more than fifty viruses and viroids (Harahagazwe et al., 2018). Among all, *Potato leafroll virus* (PLRV), *Potato Virus Y* (PVY), *Potato Virus A* (PVA), *Potato Virus S* (PVS), *Potato Virus M* (PVM), *Potato Virus X* (PVX), *Potato aucuba mosaic virus, Potato mop-top virus*, and *Alfalfa mosaic virus* (AMV) were reported to have significant damage potential. Different aphid species, PVY strain, and infesting host range is detailed in Table 1.

**Table 1:** Aphid species that transmit PVY and their host range.

**Sl. No**.	**PVY strain**	**Host range**	**Aphid species**	**Country**	**Reference**
1	PVY^O − O5^ and PVY ^NA − N^	Potato	*Aphis fabae*	South America	Fuentes et al., 2019
2	PVY^O^	Potato	*Aphis fabae, Acyrthosiphon pisum, Myzus persicae*	World wide	Lorenzen et al., 2006
3	PVY^U^	Potato	NA	Brazil	Janzac et al., 2015
4	PVY^C^	Pepper, potato	NA	NA	Kerlan and Moury, 2008
5	PVY^N:O^	Potato, pepper	*Aphis nasturtii*	North America, Austrelia	Singh et al., 2003; Piche et al., 2004; Crosslin et al., 2005; Rodriguez-Rodriguez et al., 2020
6	PVY ^N^ ^Wilga^	Tobacco	*Myzus ascaionicus, Sitobion graminum*	Europe	Verbeek et al., 2010; Crosslin, 2013
7	PVY^NTN^	Potato	*Myzus ascaionicus, Aphis fabae*	Switzerland	Boquel et al., 2011; Dupuis et al., 2019
8	PVY^N^	Potato, Tobacco	*Myzus persicae, Metopolophium albidum, Macrosiphum euphorbiae, Hyperomyzus lactucae*	North America, Africa, New Zealand	Fox et al., 2017

The vegetative method of propagation increases the susceptibility of potatoes to plant pathogens. Among different viruses, PVY (*Potyviridae*, containing 160 species) (Wylie et al., 2017; ICTV Report on Virus Classification Taxon Nomenclature, 2020) is ranked fifth across the world's top ten most important plant viruses (Scholthof et al., 2011) and considered to be economically harmful (Valkonen, 2007). Other Solanaceous hosts of PVY include tomato (Noha et al., 2018), pepper (Moodley et al., 2019), and tobacco (Guo et al., 2017). PVY is genetically composed of RNA (positive-strand of 9.7 kb in size) containing around 3,061 amino acids (Kreuze et al., 2020), and it occurs in almost all potato growing areas. PVY has been reported to be vertically transmitted from infected plants to offspring sexually through seeds or vegetatively through tubers, and from infected plants to the other plants of the same generation (horizontally), either by aphids or by mechanical contacts of adjacent plant parts (Da Silva et al., 2020). Additionally, plants grown from PVY infected tubers have a slow growth rate, resulting in seed degeneration that leads to a qualitative and quantitative reduction in potato yield (Hegde et al., 2021). Different aphid species have been reported to transmit PVY in a non-circulative and non-persistence manner with differential transmission efficiency (Lacomme et al., 2017). Infection by PVY on potatoes causes mild to severe symptoms including mottling, mosaics, yellows, rogues, necrosis, leaf malformation, plant defoliation, and potato tuber necrotic ring spot diseases (PTNRD) (Nie et al., 2012). Depending on environmental conditions and genetic diversities of both PVY (the virus strain) and the concerned host plant, the intensity, and type of these symptoms were also observed to vary (Lacomme et al., 2017). The severe economic impact of PVY on potato across different countries were reported as 30–40 % in India, 16.5% in Ireland, 34% in Canada, 37% in Kenya, 40–44% in Poland and the USA, and about 50% in China (Gray et al., 2010; Wang et al., 2011; Were et al., 2013; Hasiow-Jaroszewska et al., 2014; Hutton et al., 2015; Jailani et al., 2017). Despite several management strategies with insecticides and host plant resistance for aphid management, it's incurable under field conditions. Effective prophylactic measures are focused on preventing or slowing down the virus spread by using resistant cultivars (Dupuis et al., 2019) of healthy plant material (Funke et al., 2017) or through the eradication of diseased plants from the field. Millions of dollars have been invested in vector management worldwide specifically through chemical control, yet, the development of resistance in the aphid population and indiscriminate use of insecticides have had severely detrimental effects on the environment as well as on beneficial organisms. Thus, it is crucial to understand the interactions between the aphids and PVY and to disrupt the inter-relationship among them which can be a unique approach to managing the virus-vector complex. Among the prophylactic controls, early, sensitive, and specific detection through phytosanitary actions are the most effective (Rubio et al., 2020). Although molecular-based diagnostic techniques can be an effective identification tool, the large genetic diversity among viral species is considered to be a severe constraint (Glais et al., 2017). Although there is wide research on PVY transmission, diversity, detection, and molecular aspects available, the present review provides a comprehensive insight into potato viruses with a focus on PVY and their characteristic mode of transmission, genome organization, protein function, genetic diversity, molecular and bioinformatics aspects of transmission by aphids, methods used in detection and characterization, transcriptomics level of response, identification and monitoring of virus transmission, and vector control strategies.

## PVY and their characteristic mode of transmission

Aphid infestation and PVY infections decrease the quality and quantity of potato yields (Scholthof et al., 2011) and result in the degeneration of seed tubers that hinders potato cropping and seed production. Different species complexes of aphids were found to transmit PVY in a non-circulative, non-persistent (NCNP) manner with differential relative transmission efficiency (Lacomme et al., 2017; Da Silva et al., 2020; Hegde et al., 2021). The most crucial factors influencing PVY transmission are: (1) the acquisition source of the virus; (2) the transmissibility range of virus strains by the aphids/mechanical agents; (3) the reception of the viral strain through the aphid vector from the inoculated plants; (4) the growth and development of the aphid on the host plants, followed by inoculation, acquisition, and transmission of PVY (Bosquee et al., 2018); and finally, (5) the aphid vectors transmitting PVY. Furthermore, PVY transmission is also influenced by temperature, (relative) humidity (Chung et al., 2016), and atmospheric gas concentration (Dáder et al., 2016; Bosquee et al., 2018). These environmental factors could change the vector-virus interaction, and thus the aphid behavior, plant susceptibility, and/or virus replication in PlantScan would ultimately be changed (Van Munster, 2020). So, knowledge of the factors affecting the transmission efficiency of aphid vectors is crucial in understanding epidemiology and improving the control of viral diseases.

### Process of acquisition and transmission through aphid

The feeding and transmission process comprises systematic events from starving, acquisition accession period (AAP), inoculation accession period (IAP), and virus retention inside the vector body. Starving before feeding and inoculation increase the non-persistent viral transmission efficiency in aphids (Powell, 1993). Starving enhances the feeding behavior and appetite of aphids (Jimenez et al., 2017). Diverse viral transmission studies using starved aphids (for 2–3 h) were reported to increase the transmission efficiency and decrease the AAP and IAP (Fereres and Moreno, 2009). Optimally, the aphids were observed to have an AAP and IAP of 1–30 s. The virulence of *Myzus persicae* remains from 2 to 4 h depending on the number of host plants visited, feeding duration, and the retention period. As the non-persistent viral transmission is of lower efficiency and less host specific (Pirone and Harris, 1977), the feeding behavior of aphids, compatibility with the helper component, and/or availability of receptors are major determinants (Uzest et al., 2007) of PVY transmission (Nanayakkara et al., 2012). These parameters, though, affect aphid feeding behavior, transmission efficiency, and virus epidemiology, yet, molecular properties of the aphid determines the final transmission and severity of PVY.

### Host range

A wide diversity of plants have been listed as natural PVY hosts and comprise 495 species of plants from 72 genera belonging to 31 families including edible crops such as potato, tomato, pepper, and eggplant, in addition to 211 species within 9 genera of the Solanaceae family. Additionally, Amaranthaceae, Fabaceae, Chenopodiaceae, Compositae, and Brassicaceae were also reported to be host species of PVY (Kaliciak and Syller, 2009). The host diversity was reported to indicate differential evolutionary routes by natural selection pressure, together with mutation and recombination (Moury and Desbiez, 2020).

## Genome organization and proteins

Like other potyviruses, PVY is filamentous having flexuous virus particles (700 nm × 11–13 nm) and a single-stranded positive sensed RNA genome (+ssRNA), encapsulated with the coat protein of 2,000 units of the same monomer (30 kDa). The RNA genome contains 9,700 nucleotides ending with a viral protein genome-linked (VPg) at the 5′ end and a poly-A tail at the 3′ end. Furthermore, VPg is a multifunctional protein at the 5′ terminus of +RNA and acts mainly as a primer during RNA synthesis in a variety of +ssRNA viruses, including potyviridae where the poly-A in 3′ of RNA terminus (mRNA and +RNA viruses) that has only adenine bases and is involved in gene expression. The PVY genome is reported to contain two open reading frames (ORF), including the main ORF that is translated to a large polyprotein which ultimately terminates in 10 functional proteins, and the small ORF, otherwise known as Pretty Interesting Potyviridae ORF (P3N-PIPO or PIPO), that is a +2 frameshift within the main ORF and is translated into another protein of smaller size (Quenouille et al., 2013; Valli et al., 2018).

### Genetic diversity and strains

Genetic variation in RNA viruses including PVY is known as quasispecies and these are the results of mutations, recombination, and selection pressures from hosts, viruses, and importantly virus-vector interactions (Domingo and Perales, 2019). Shortly after penetration into the host cell, the functional mRNA of the PVY genome is translated and this process lacks all kinds of proofreading activity (Elena et al., 2011), which is a major reason for higher mutation and genetic diversity of RNA viruses, especially PVY (Wolf et al., 2018). Higher mutation rates along with extreme virus diversity make the PVY more adaptable to environmental as well as host-specific conditions as compared with DNA viruses (Duffy, 2018). Random genetic drift that occurs in the process of horizontal transmission by the plant sap suckers leads to a reduction in virus diversity and has immense importance in viral evolution (Betancourt et al., 2008). Natural resistance gene pools have been targeted for breeding PVY resistant cultivars, as the intensive potato cropping along with injudicious pesticide application have led to crop failure. These factors altogether contributed to the mutant emergence and strain diversity in PVY (Funke et al., 2017; Dupuis et al., 2019). Further diverse PVY genetic strain development could be attributed to mutation, recombination, reassortment in the multipartite virus, and selection pressure from host, virus, and vector interactions (Kutnjak et al., 2015; Domingo and Perales, 2019). Although resistance genes are being deployed against PVY, they are capable of breaking the resistance and creating new genetic strains by interacting with PVY populations in highly divergent ways, exhibiting rapid co-evolution or stable associations (Karasev and Gray, 2013; Quenouille et al., 2013).

### Molecular basis of PVY transmission

#### Non-persistent transmission of PVY by aphids

**Figure 1:**
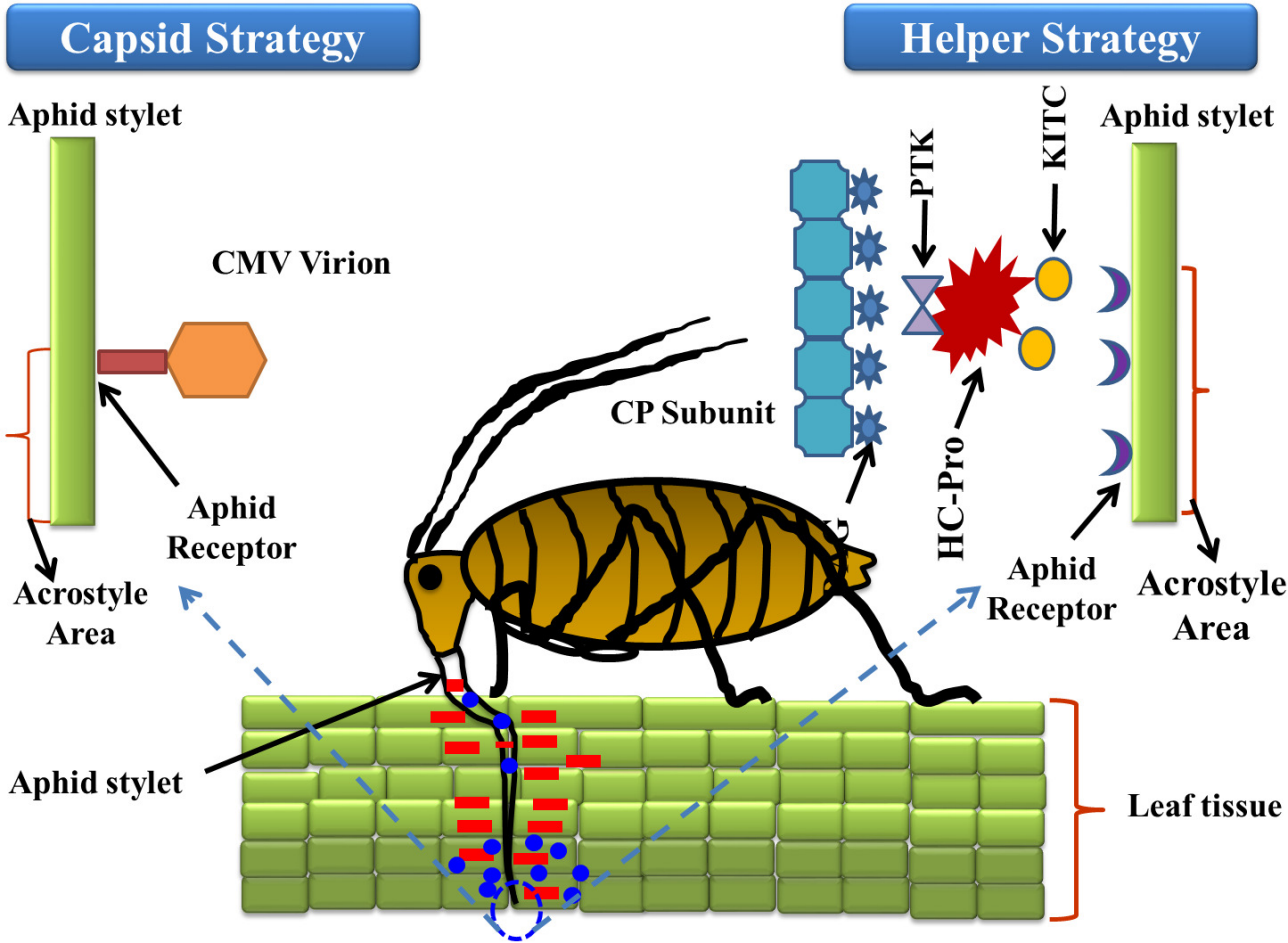
In the former, coat protein (CP) of virus interact directly with the binding sites (receptors) present in the stylet of aphid; in later, an additional non-structural protein namely HC-Pro (helper component proteinase) induces the binding between CP and aphid receptor component, thus creates a reversible “molecular bridge” that leads to effective infection by the aphid vector. In relation to aphid transmission, HC-Pro N-terminal domain (KITC—Lysine/Isoleucine/Threonine/Cysteine) is involved in specific binding to an aphid's stylet tip (acrostyle); while it's C-terminal domain (PTK-Proline/Threonine/Lysine) is involved either directly or indirectly in HC-Pro binding to the DAG motif (Aspartic acid/Alanine/Glycine) at the CP N-terminus.

**Figure 2:**
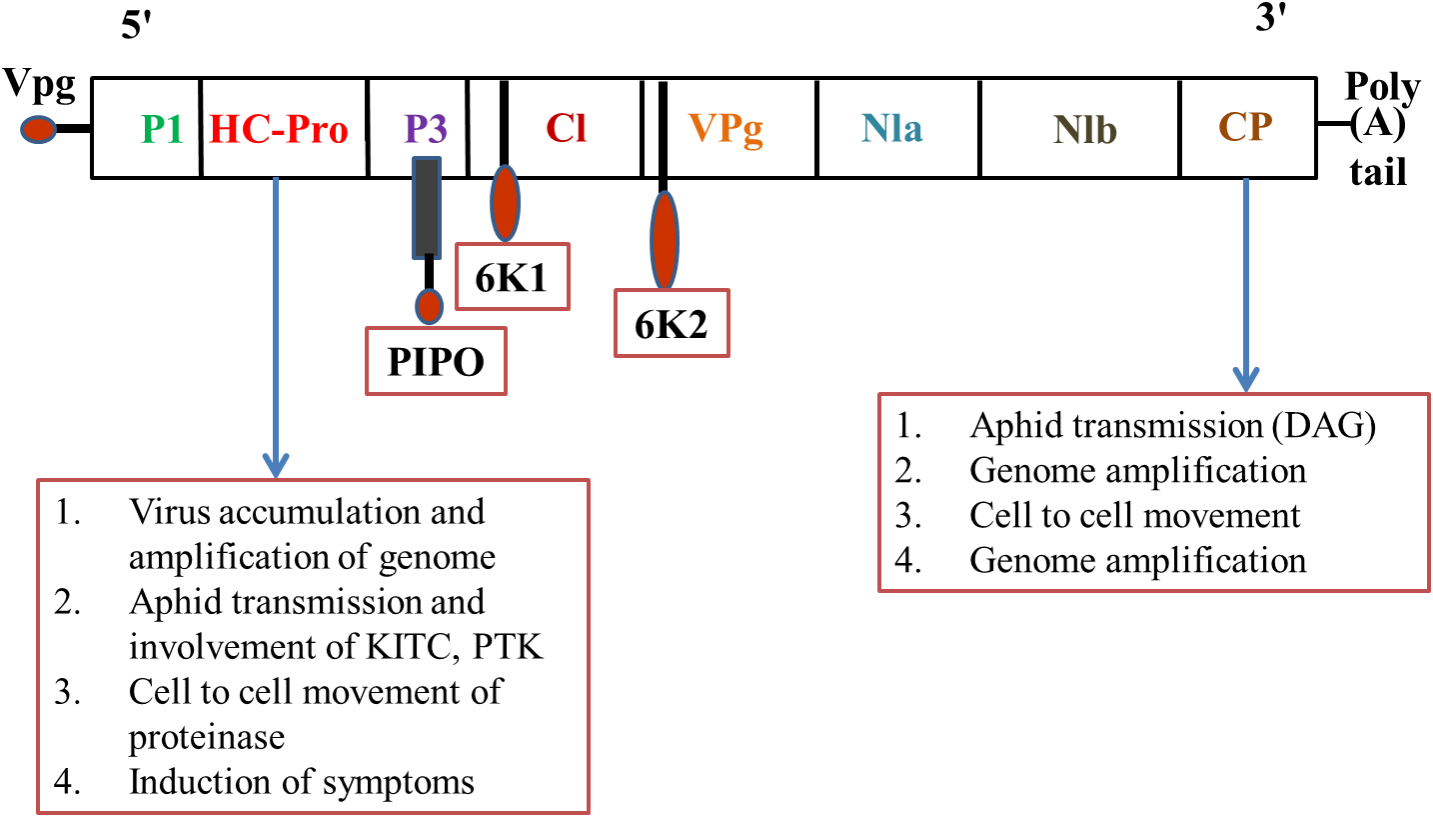
Potyvirus proteins and their functions (Mishra et al., 2014). P1 Proteinase—Cell to cell movement; HC-Pro— Aphid mediated transmission, cell to cell movement; P3—Role in replication; CI—Genome replication (RNA helicase), membrane attachment, ATPase activity, cell to cell movement; VPg—Genome replication (Primer for initiation of RNA synthesis); NIa—Major Proteinase; NIb—Genome replication (RNA-dependent RNA polymerase [RdRp]); CP—RNA encapsidation, involved in vector transmission, cell to cell movement; 6K1, 6K2—Possible roles in RNA replication, regulatory function, inhibiting NIa nuclear translocation.

Transmission of plant viruses may occur in four ways, i.e., non-persistent; semi-persistent; persistent circulative; and persistent propagative, depending on the localization of the vector, the time required for virus acquisition along with retention, and potential transmission of the viral load (Sylvester, 1980). The non-persistent (stylet borne) viruses have a shorter acquisition period, from seconds to a few minutes, while semi-persistent viruses require several hours for acquisition, and once inoculated the vector loses its transmission capacity until the next acquisition (Dietzgen et al., 2016). Over 20 species of aphids were potential transmitters of PVY and colonize and multiply on solanaceous hosts (Boiteau et al., 1988). The complex mechanism of virus-vector association requires a continuum of suitable molecular *cum* biochemical interactions with the host plant for successful establishment and transmission. The strategies involved in virus-vector association can be broadly differentiated into two categories, i.e., capsid strategy and helper strategy (Figure 1). In the former, the coat protein (CP) of the virus has been observed to interact directly with the binding sites (receptors) present in the stylet of the aphid; in the latter mechanism, an additional non-structural protein namely HC-Pro (helper component proteinase) induces the binding between CP and the aphid receptor component, thus creating a reversible “molecular bridge” that leads to effective infection by the aphid vector. The binding process is governed by multiple factors such as environmental factors, aphid species, aphid-virus interactions, the site of binding, the molecular configuration of the binding site, and the viral protein structure. The PVY genome comprises dsRNA of nearly 10 kb size that on further polyadenylation forms a single open reading frame (ORF) of large polyprotein of 340–368 kDa size. This ORF, upon activation by the viral proteases, produces 10 diverse proteins of differential functions including: P1 (protein 1 protease), NIa-Pro (nuclear inclusion A protease), HC-Pro; P3 (protein 3), 6K1, and 6K2 (six kilodalton peptides); CI (cytoplasmic inclusion); NIb (nuclear inclusion B/RNA-dependent RNA polymerase); CP (Adams et al., 2005). Furthermore, in the transmission of cucumoviruses such as the cucumber mosaic virus (CMV), the viral CP alone binds to the aphid stylet to induce aphid-mediated transmission (Gadhave et al., 2020), while in PVY transmission the non-structural VPg (viral protein genome-linked) was reported to have a covalent association with the 50-terminal in the Helper strategy (Adams et al., 2005). The PVY like potyviruses require a more intricate association between CP and HC-Pro to establish a successful association between the virus and aphid stylet (Valli et al., 2018). HC-Pro is the key regulator as it contains three overlapping regions governing multiple functions like interactions among plants, aphids, and virions, amplification of the potyvirus in the aphid vector, suppression of the gene silencing, systemic movement of the viral load within the plant phloem, development of symptoms, and ultimately the cleavage and activation of proteins through protease activity (Anandalakshmi et al., 1998). Genome-wide variation analysis of potyviruses suggests that these contain diverse hypervariable areas in key parts of the genome such as P1, HC-Pro, P3, VPg, NIb, CP, NIb-CP junction, and NIa protease (Deepti et al., 2019) that enrich the virus mutationally and are helpful in adapting diverse hosts. Phylogenetic analysis of CP sequences (from 176 potyviruses) further confirms the role of HC-Pro in direct/indirect interaction between virions and aphid stylets, which enables retention of virus, and further inoculation (Valli et al., 2018). Thus, a mutation in either HC-Pro or CP of potyviruses can alter aphid transmissibility. The viral genome undergoes autoproteolytic reactions, catalyzed by two proteinases, P1 and HC-Pro, at the respective C termini (Verchot et al., 1991), while the remaining cleavage reactions are catalyzed by the NIa-Pro, an evolutionary homolog of the picornavirus 3C proteinase (Carrington and Dougherty, 1987a,b). The proteins are multifunctional (Mahajan et al., 1996) and their conserved regions include the HC-Pro and NIb, while the variable regions include P1, P3, and CP. It has been reported that P3 exhibits low homology between species, with variations observed between P3 proteins of different potyviruses (Aleman-Verdaguer et al., 1997). P3, the conserved protein was observed to play an important role in the virus' functioning, mostly associated with the cytoplasm of potyvirus-infected cells. A highly conserved DAG motif of CP interacts with either the PTK motif or its functionally similar motif/s (C-terminus) of HC-Pro (Huet et al., 1994) to provide binding of HC-Pro to the coat protein of virions. Furthermore, another equivalent motif, i.e., the KITC motif (N-terminus), was reported to be critical for virus retention in the stylets of aphids (Huet et al., 1994). Both the interactions in the C-terminus and N-terminus are essential for the potyvirus transmission by aphids and successful colonization on the solanaceous hosts (Blanc et al., 1997). Further studies also confirmed that certain genera of *Potyviridae* like rymovirus, Poacevirus, and tritimovirus are not transmitted by aphids due to a lack of suitable amino acid motifs for proper binding (Wylie et al., 2017), so they are transmitted in a semi-persistent manner by the eriophyid mites. Another virus, *Rose yellow mosaic virus* (RoYMV) from the monotypic genus Roymovirus was reported to lack the DAG motif in the CP, and the substituted HC-Pro motifs PTK and KITC by the C-2x-C motif at the N-terminus, favors transmission by the eriophyid mite (Wylie et al., 2017). Regarding another virus of the bevemovirus genus, i.e., *Bellflower venial mottle virus* (BVMV), the DTG motif similar to DAG is found near the N-terminus of CP, but it lacks the PTK and KITC motifs, so it is non-transmissible by the aphid vectors (Wylie et al., 2017). The detailed structure of the PVY coat protein is depicted in Figure 2. Further details of individual proteins along with their genome size and functions are detailed in Table 2.

**Table 2:** Potyvirus proteins and their functions (Hu et al., 2011; Hillung et al., 2013; Revers and Garcia, 2015; Lacomme et al., 2017; Valli et al., 2018).

**Sl. No**.	**Protein**	**Function**
1	P1	Accessory factor for virus amplification, stabilizes CP, stimulate HC-pro silencing suppression, Binds RNA.
2	HC-Pro	Helper factor for aphid transmission, RNA silencing suppression, Enhancement of yield of virus particles, HC proteinase, resistance breaking, virus movement.
3	P3	Virus amplification, host adaptation and pathogenicity, resistance breaking.
4	P3N-PIPO	Viral cell to cell movement.
5	6K1	Modulation of P3 activity? Exact function is unknown. Role in potyviral infection.
6	CI	RNA helicase, RNA replication, pinwheel formation (forms the cylindrical cytoplasmic inclusions), virus movement, cell to cell movement, binds RNA.
7	6K2	Membrane vesicles proliferation, membrane targeting to ER-types membrane, replication, systemic movement.
8	VPg	Genome-linked protein, primer of RNA replication, RNA translation, cell-to-cell and systemic virus movement, nuclear inclusion, nuclear transport, resistance breaking, symptom modification.
9	NIa	Cysteine proteinase, DNAse, small nuclear inclusion protein A, serine-like proteinase activity: cleave P3-6K1-CI-6K2-Nia-Nib-CP, PrimeRNA synthesis, required for systemic infection, nuclear transport, cell-to-cell and systemic movement, resistance breaking.
10	NIb	Large nuclear inclusion protein B, RNA replicase, Viral replication, nuclear transport, Symptom modification.
11	CP	Coat protein (protection of genomic RNA), cell-tocell and systemic movement, aphid transmission, virus encapsidation and virus assembly, regulation of viral RNA amplification, seed transmission, symptom modification, translation.

#### PVY molecular responses in plants: A spectrum of results from sensitivity to resistance

The intricate relationship between host plants and viruses involves the interaction of numerous systems, including (1) viral regulation of host components essential for effective infection, (2) plant defensive mechanisms, and (3) the counter-defense mechanisms of viruses to evade plant defenses. The inter-relationship among these factors is a suitable determinant of reactions among the compatible interaction of virus or incompatible host interaction. In response to aphid infestation, susceptible plants develop a diverse local and systemic infection, while leaves of tolerant plants undergo mild symptoms (Cooper and Jones, 1984). Replication of the virus and further spreading are reported to be restricted in the defense response by an incompatible reaction. Resistance to PVY can be categorized into three types: extreme resistance (ER), hypersensitive reaction (HR), and tolerance. The presence of susceptible genes (S-genes) along with apparent signs and minimal necrotic lesions enable the plants to be extremely resistant; meanwhile, local necrotic lesions were observed in hypersensitive reactions (HR), and plants that overcome the aphid infestation and viral transmission and grow adequately are considered to be tolerant (Valkonen, 2015). Although, ER, HR, tolerance, and susceptible responses were well-documented in potato, there have been no reports of natural S-gene mediated resistance to date. In the event of ER response, potato plants exhibit no symptoms or very minimal microscopic lesions in certain genotypes (Valkonen and Palohuhta, 1996). Suppression of virus multiplication in infected potato cells and limited cell-to-cell movement was observed in ER response (Seo et al., 2014). The first R gene (Ry_sto_ gene), which confers ER response to PVY in potato, was identified and functionally described (Grech-Baran et al., 2019), and it was reported to encode an intracellular nucleotide-binding leucine-rich repeat (NLR) receptor with an N-terminal Toll/interleukin-1 receptor (TIR) domain (TIR-NLR) that imparts resistance to PVY and PVA in potato and tobacco plants. Another R gene against PVY was identified from pepper (*Capsicum annuum*) (Kim et al., 2017). Another dominant gene, Pvr4, encodes a protein of the LRR receptor with an N-terminal coiled-coil domain (CC-LRR) and imparts ER (Dogimont et al., 1996). Some key downstream signaling processes like activation of resistance and immunity are mediated by TIR-NLRs *via* the lipase-like protein Enhanced Disease Susceptibility 1 (EDS1) (Aarts et al., 1998) and CC-NLR protein N requirement gene 1 (NRG1) (Castel et al., 2019; Grech-Baran et al., 2019). Among diverse qualitative changes in resistant genotype PW363, stress-responsive proteins were found to be the most prevalent (Szajko et al., 2018).

**Figure 3:**
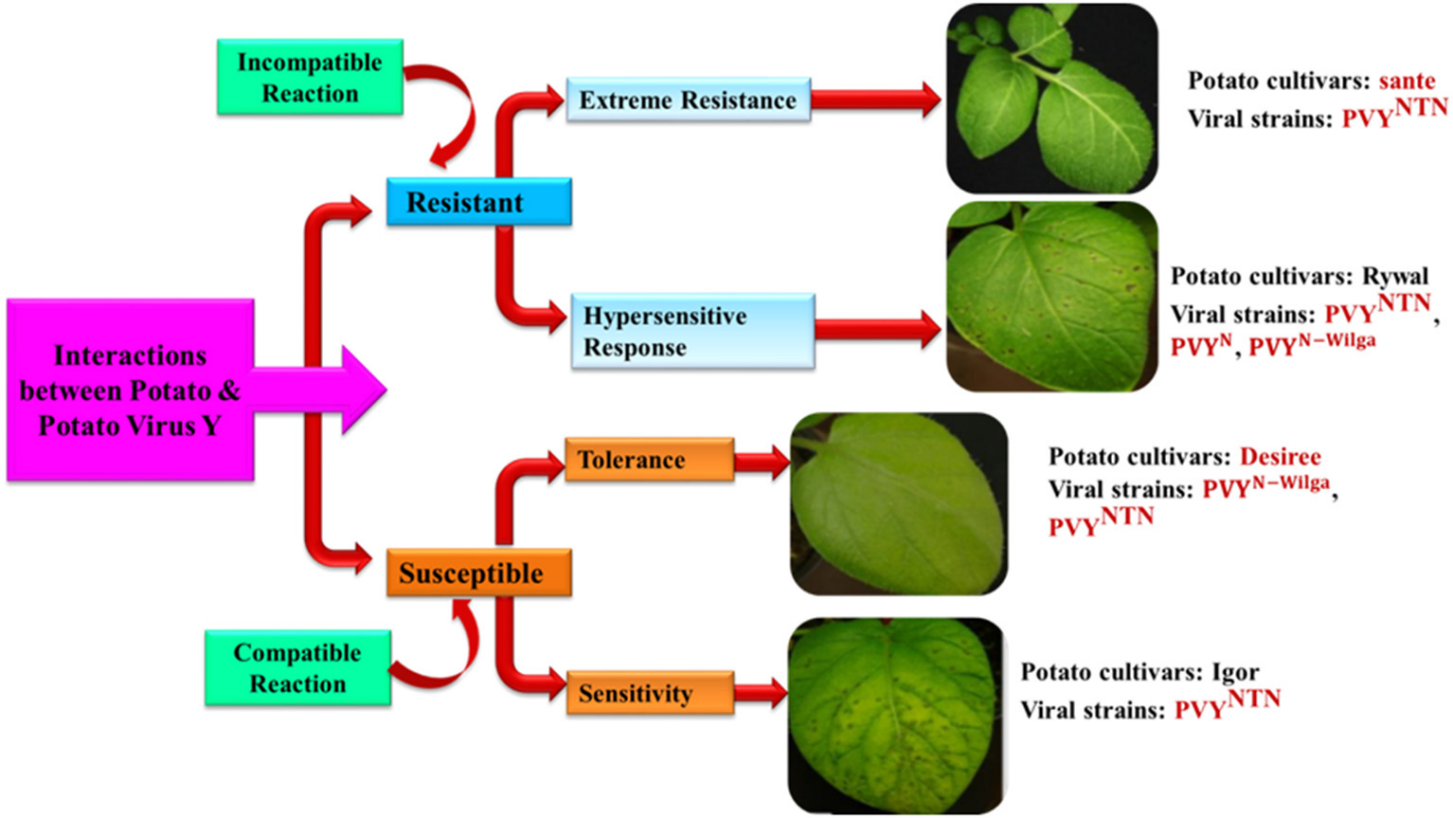
Interaction between potato and *Potato Virus Y* depicting incompatible and compatible resistance and susceptibility reaction in different potato varieties. Differential reactions (Extreme resistance, hypersensitive resistance, tolerance and sensitivity) are determined by the host genotype, viral strain, and environmental factors, and appear as varied responses in terms of virus replication and disease morphologies. Images representing symptoms on leaves 6 days after inoculation in selected cultivars of potato in optimum environment condition (Modified from Baebler et al., 2020).

HR resistance is distinguished from ER by predominant localized tissue necrosis caused by programmed cell death (PCD). This HR was introduced into potato from wild relatives, Ny, Nc, and Nz, imparting resistance to PVY^O^ (ordinary strains), PVY^C^ (C strains), and PVY^Z^ (Z strains), respectively (Valkonen, 2015). The defensive reaction initiated from the synthesis of reactive oxygen species (ROS) followed by the production of hydrogen peroxide and the activation of numerous genes (Balint-Kurti, 2019) in the Ny-1-mediated resistance of cv. Rywal, 1-day post-inoculation (dpi) (Baebler et al., 2014). HR-mediated resistance is governed by Mitogen-activated protein kinase kinase 6 (MKK6), and downstream targets MAPK4 (Mitogen-activated protein kinase 4), MAPK6, and MAPK13 (Lazar et al., 2014). Inhibition of MKK6 was demonstrated to enhance PVY concentrations in infected potato plants, indicating its key role in potato virus immunity (Dobnik et al., 2016). The host—PVY interaction is reported to be determined by diverse factors, namely the host genotypes, viral strains, and environmental circumstances, and they appear as various responses in terms of the multiplication of viruses and the progression of disease symptoms. The details of symptoms appearing on the inoculated leaves 6 dpi in selected potato cultivars in an optimal environment are given in Figure 3 (Baebler et al., 2020).

The results of the interaction among host—PVY in the same potato genotype change based on PVY strain, as demonstrated by PVY^N^ and PVY^NTN^ (cvs. Igor and Nadine) (Kogovšek et al., 2010) and PVY^N − Wilga^ and PVY^NTN^ in cv. Etola (Yin et al., 2017). In contrast, the manifestation of symptoms in HR response to PVY^N605^-GFP, PVY^NTN^, and PVY^N − Wilga^ did not vary in the cultivar Rywal (Lukan et al., 2020). Numerous abiotic factors also influence the outcome of the interaction (Makarova et al., 2018) and, in combination, infections with different viruses. The molecular mechanisms underlying those results are critical for resistance breeding without growing trade-offs and agronomic practice adaptability. Plant molecular responses to PVY were compared and connected to recognized molecular pathways of plant defense. The viral effector protein produced upon aphid infestation is recognized by the R-gene, which activates an array of defense responses, i.e., MAPK, Salicylic Acid (SA), and Reactive Oxygen Species (ROS). The resistance process is activated by the pathogenesis-related genes (PR 1) and β-1, and 3-glucanase gene (BGLU), which results in the strengthening of the cell wall, accumulation, and deposition of callose and photosynthesis transient induction in case of HR- mediated Resistance. Similarly, in case of ER, N requirement gene 1 (NRG 1) is needed for biochemical activation downstream and it depends on Enhanced Disease Susceptibility (EDS 1). Finally, BGLU is involved in the viral defense. Tolerant interactions can occur as a result of a failure to recognize an R-gene (e.g., TPN1) or cochaperone (e.g., Hop/Sti1) essential for viral replication. SA has a crucial function in the development of the tolerance reaction in plants against PVY. There is no involvement of ROS, PR1, BGLU, or callose deposition, but temporary photosynthetic activation and metabolite reallocation were detected. The viral effector protein is identified by an ineffective R gene in sensitive interactions that further results in a delayed or altered downstream production of ROS, SA, and jasmonic acid/ethylene (JA/ET) signaling. Moreover, the same viral protein leads to the activation of proteins like PR1 and BGLU, which governs the virus-vector interaction. However, in this case, no cell wall strengthening or callose deposition was observed.

#### Transcriptome and small RNA profiling of PVY infected potato cultivars

The damage caused by PVY along with PLRV and other viruses has been observed to vary from 10% to entire crop loss (Warren et al., 2005). The virus infection can be measured by the transcriptome analysis by analyzing the highly regulated genes (upregulated and downregulated) in potato (host) -virus complexes. The positive log fold changes were governed by upregulated genes, while the negative log fold changes were by downregulated genes. PVY-induced altered-gene expression in the potato cultivars was estimated. The total RNA from both viruliferous and healthy potato cultivars was extracted and converted to complementary DNA (cDNA) by Real Time PCR. RNA-Seq libraries for all samples were prepared and sequencing was done in HiSEQ 4000 lane with paired-end chemistry of 150 bp. The tagged cDNA libraries were pooled in equal ratios and used for 2 × 150 bp end sequencing. Flow cells for Illumina HiSeq 4000 instruments were loaded with Illumina clusters and sequencing was performed. Read filtration and assessment of differential gene expression were done with annotation and functional enrichment analysis of differentially expressed genes (DEGs). Highly up- and downregulated DEGs of potato cultivars were selected to validate the differential gene expression data by RNA-Seq analysis. The C_T_ values provided by quantitative Reverse Transcription PCR (qRT-PCR) for both endogenous genes and targeted genes were used to calculate the log_2_-fold changes (Livak and Schmittgen, 2001). In upregulation, the quantity of mRNA or protein production increases for viruliferous as compared to healthy crops. The total number of genes influenced by PVY infection was calculated to be highest, i.e., 5,730, in resistant potato cultivars (53 percent upregulated and 47 percent downregulated), and 4,238 in susceptible cultivars (46 percent upregulated and 54 percent downregulated). In total, 1,285 genes for bacteria, 4,591 for fungus, 4,218 for nematode, 2,356 for insects, 4,892 for cold, 3,456 for heat, 2,878 for salt, and 4,241 for drought stressors were found to have involvement in virus-vector interaction, indicating maximum involvement of differentially regulated genes in potato-PVY transmission interaction (Osmani et al., 2019).

#### Gene ontology (GO) analysis

Gene Ontology (GO) is a bioinformatic initiative undertaken to unify the representation of gene and gene product attributes across species that utilizes different gene annotations against the UniProt GO database (http://geneontology.org/). By GO enrichment analysis the altered processes and functions of the vector in response to the virus are defined. The numbers of DEGs were reported to be involved in biological-cellular-molecular functions, and biological processes can be estimated by conducting the GO enrichment analysis. Cellular components involve different organelle membranes, protein complexes, and the connecting synapses; while biological processes include DEGs governing diverse cellular processes like a response to diverse stimuli, signaling, bio-metabolic processes, biological regulation, and signal transduction. Furthermore, in the molecular functions category, DEGs are associated with diverse catalytic activity, binding, transduction, antioxidant, and transporter activities.

#### Molecular and bioinformatics interaction of host plant-aphid-PVY transmissions

The CP of PVY was examined by Moury and Simon (2011) to identify positive selection connected with trade-offs between several fitness features that have implications in adaptation processes by using the dn/ds (non-synonymous/synonymous substitution rates) ratio and numerous potential positively chosen codon were identified using technologies like PAML (Phylogenetic Analysis using Maximum Likelihood) and hyphy (Hypothesis testing using phylogenies) in PVY N clade at codon positions 25 and 68. Furthermore, a volunteer plant of potato (cv. Diacol-Capiro) was infected with PVY and the potato leaf roll virus (PLRV) (Medina Cárdenas et al., 2017). In this study, researchers observed the effectiveness of RT-PCR (RT-qPCR) and next-generation sequencing (NGS). These results were helpful in different viral strain identification in natural reservoirs like weeds and stray plants, thus can be effective for enabling integrated management of plant viruses in potato. Another multiomics investigation involving the tolerant interaction of potato with PVY was deciphered by Stare et al. (2019) using cv. Desiree. They examined the dynamism at molecular, transcriptomics, sRNAomics, degradomics, proteomics, and hormonomics stages and captured the virus-vector interaction. The measures of accumulation of virion, photosynthetic activity, and phenotyping of the symptoms were compared with its transgenic counterpart, NahG-Desiree, which cannot accumulate salicylic acid, which plays a significant role in plant defense. For each genotype and time point, they utilized the empirical Bayesian technique and identified differentially expressed genes between healthy and PVY-inoculated plants. The SEQUEST method was used to identify the proteins for the proteomics analysis and differentially abundant proteins were identified. The entire piece has been uploaded to FAIRDOMhub. Data from microarrays, sRNA-Seq, and degradome-Seq can be found in the Gene Expression Omnibus (GEO) collection at the NCBI. ProteomeXchange has proteomics data with the identifier PXD0152215 accessible. This investigation offers further clarity into the processes underlying the potato's tolerant response to viral infection and can serve as a starting point for future research on the regulation of plant immunity. Based on the VPg Gene, Mao et al. (2019) conducted research on the phylogenetic analysis of the PVY infection by following a Bayesian approach and 177 nucleotide sequences from the viral genome linked protein (VPg) gene which was reported to interact with the plant eukaryotic translation initiation factor 4E (eIF4E). The results confirmed that the VPg gene of PVY has been evolving at a rate of 5.60–10^−4^ subs/site/year, which is comparable to that of other plant-infecting RNA viruses. Additionally, they discovered a correlation between genetic variations and geographical locations, indicating that the evolution of this virus is significantly influenced by geographically linked factors or epigenetic factors. Two distinct methods were utilized to find probable recombination events in VPg sequences in order to examine the role of recombination in PVY evolution. First, using SplitsTree 4.13.1's NeighborNet algorithm, we executed a split-decomposition network analysis and got the pairwise homoplasy index (Huson, 1998). Moreover, potential recombinant and parental sequences were identified using RDP, BOOTSCAN, MAXCHI, CHIMERA, SISCAN, GENECONV, and 3SEQ type algorithms (Martin et al., 2015). The generated datasets can be further found in MK144421-MK144464, NCBI.

Deepti et al. (2019) investigated potyviruses' genome-wide variation and examined the genomic and polyprotein diversity in all species of potyvirus. The results demonstrated that the potyvirus genome is strongly under negative selection and, for the first time, that the number of locations under positive selection is correlated with the host range. Recombinant nucleotide sequences were detected using RDP4 (Martin et al., 2015) and the sequences with recombination breakpoints were evaluated using six distinct methods: RDP, GENECONV, 3SEQ, SISCAN, MAXCHI, and BOOTSCAN. MAFFT version 7.3 was used for its tree-based progressive technique to study MSA (Multiple Sequence Alignments); gaps from the alignment were eliminated using GapStrip/Squeezev2.1.0; the best-fit nucleotide and protein replacement model was selected based on the lowest Bayesian Information Criterion (BIC) by PhyML 3.0's—(Phylogenetic analysis using Maximum likelihood) Smart Model selection (Lefort et al., 2017). The genomic or polyprotein sequence alignment obtained from MAFFT was utilized for SNP or SAP identification in the polymorphic study of each virus species. In a variant call format (VCF), the extraction of the type and location of each replacement and hierarchical clustering for sequence variation groups were also carried out (Danecek et al., 2011; Wickham et al., 2013). The structural foundation for the multitasking character of the PVY coat protein was explored by Kezar et al. (2019). The near-atomic structure of PVY's flexuous virions was determined using cryoelectron microscopy, which also revealed a previously unrecognized lumenal interaction between prolonged C-terminal portions of the coat protein units and viral RNA. These structures provided the initial proof of the flexibility of the amino- and carboxyl-terminal regions of the coat protein. They demonstrated their function in PVY infectivity by mutational analysis and in planta tests, and they provided an explanation for the coat protein's (CP) capacity to carry out numerous biological functions, thus reconfirming the importance of CP in aphid mediated viral transmission. Da Silva et al. (2020) investigated how different transmission methods have differential effects on the PVY transmission, and impact on population structure by using a deep sequencing method in plant parts like leaves and tubers of three PVY strains transmitted both by horizontal (aphid and mechanical) and vertical (by tubers) modes. The findings suggest a crucial influence of virus transmission methods on the within-plant diversity of virus populations and offer quantitative fundamental information on transmission mediated virus diversity in plants, where various transmission techniques are anticipated to influence the structure of a virus population and subsequently its evolution. Using R v3.6.1 to plot the changes in the frequency of each SNP in the viral population, structures were identified and SNP frequency trajectories were grouped into heat maps for each lineage using the Heatmap.2 toolkit after eliminating SNPs that occurred at a frequency of at least 10% in at least two different lineages to conduct a more thorough search for potentially positively selected SNPs. Another study by Tiwari et al. (2021) on the genome sequencing of the potato virus vector foxglove aphid (*Aulacorthum solani* Kaltenbach) sheds light on the virulence genes. In all, 16,610 genes out of 22,021 predicted genes had putative roles associated with other aphids, primarily *Myzus persicae, Acyrthosiphon pisum*, and *Diuraphis noxia*. Insecticide resistant genes, virus transmission genes, transcription factors, and mitochondrial genes were among the virulence genes they discovered. Other virulence genes included those for defense and detoxification, salivary genes, and chemoreceptors. Additionally, analysis of the GO and KEGG pathways revealed that genes were enriched mostly for molecular function and signal transduction, respectively. A phylogenetic examination of 12 aphid species demonstrated genetic divergence, and *A. solani* is closely linked to *M. persicae*.

## Identification and monitoring of virus transmission

**Figure 4:**
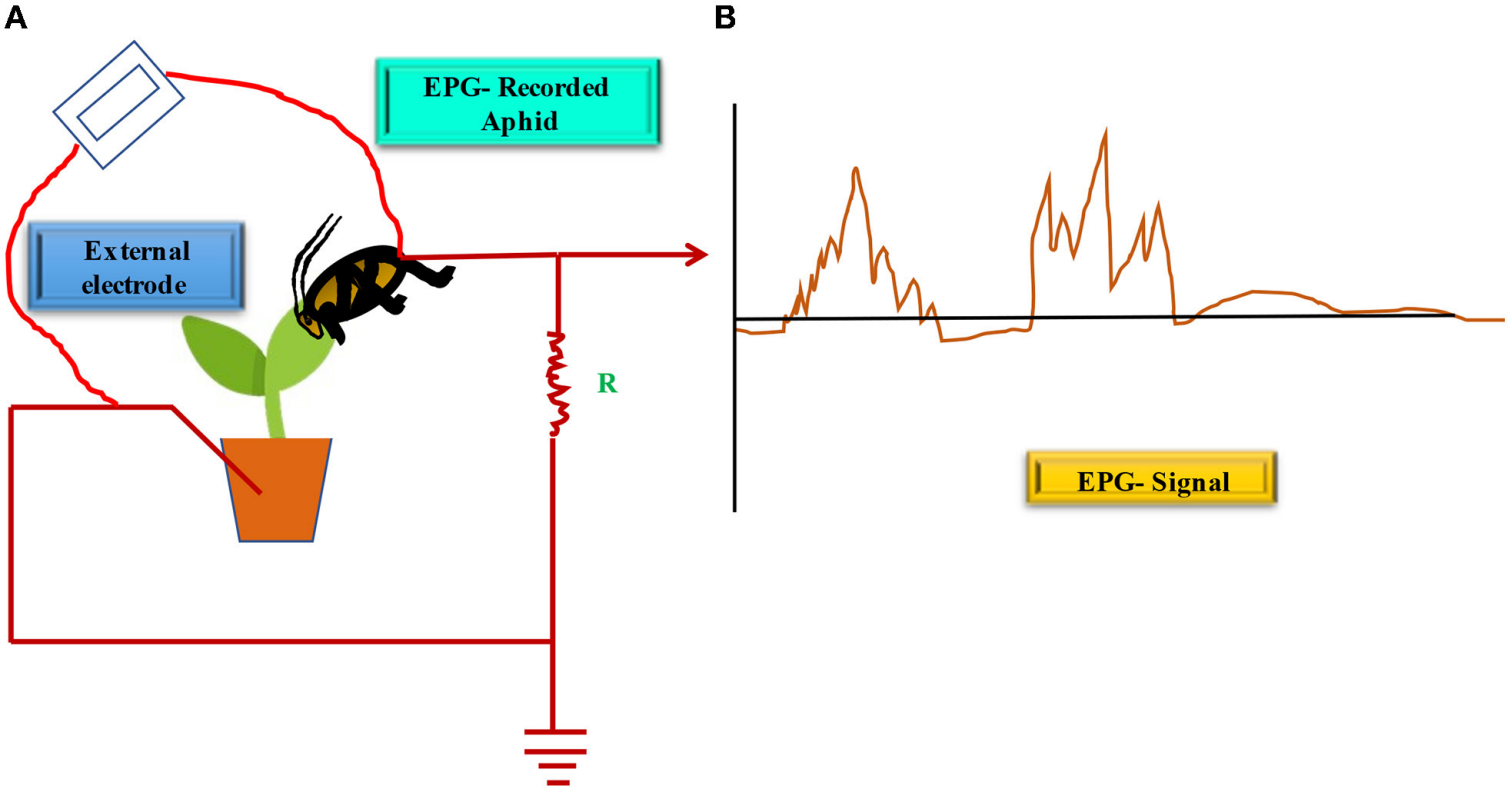
EPG studies on potato-aphid interactions showing PVY transmission. **(A)** Depicts EPG system set-up with waveform pattern, **(B)** Represents typical waveforms corresponding to different feeding phase and several potential drop (pd) phases are depicted according to different feeding functions. (Adopted from Martin et al., 1997).

Myriad approaches are possible to demonstrate that a plant virus is transmitted by a certain vector, depending on the virus, vector, transmission modes, and accessible instruments. In brief, vector transmission bioassays have indeed been conducted to investigate vector affinity, vector recognition and performance, and vector transmission control for various plant viruses. Bioassays are the most ancient way of studying viruses, and they provide vital information on epidemiology and management strategies (Dijkstra and Jager, 1998). The feeding of aphids (and/or other sucking insects) on a synthetic diet using parafilm is a commonly used bioassay in viral transmission research. The electrical penetration graph (EPG) is a tool used by biologists to explore the interactions between insects (thrips, aphids, and leafhoppers) and host plants. It was invented and revised by Mclean and Kinsey (1964) and Tjallingii (1978, 1988). The EPG system utilizes insects (sucking insects) and plants as electric circuit modules, recording electric currents once the insects begin feeding (Figure 4). During intracellular salivation, the EPG signal contains three unique sub-phases: II-1, II-2, and II-3, that are related to the acquiring (II-3) and infection (II-1) of NCNP viruses (Martin et al., 1997; Powell, 2005). This (EPG) approach was used to determine unique AAP for aphid species and to explain the transmission processes (Powell, 2005; Boquel et al., 2011). Using the EPG equipment, Boquel et al. (2011) evaluated the time between aphid placement on the plant and the first probe, as well as the AAP for a few aphids and PVY. Transmission Electron Microscopy (TEM) has also expanded the identification and diagnosis of plant viruses (Ammar et al., 1994). PVY and other non-circulative, non-persistent potyviruses were found in aphid stylets (Wang et al., 1996). The bridge theory and the role of HC were also discussed. Immunological detection methods were by far the most popular detection methods prior to the advent of molecular approaches, and they are still the preferred method in routine viral detection testing. Since 1978, the enzyme-linked immunosorbent assay (ELISA) (Clark and Adams, 1977) has been used in various investigations to identify plant viruses, including PVY, in the bodies of vectors. It is possible to detect plant viruses using a variety of molecular methods, including polymerase chain reaction (PCR). A next generation sequencing (NGS) technology provides a number of opportunities for detecting, identifying, quantifying, and studying viruses, viroids, and microbes, as well as their ecology, epidemiology, replication, and transcription (Cao et al., 2017; Rubio et al., 2020). Zhang et al. (2013) detected PVY in stylets as a way of determining vector virus transmission capacity. They observed PVY in 38% of *M. persicae* individual stylets, which is similar to PVY transmission by *M. persicae*, however, the information on transmission efficiency came from another study by Moreno et al. (2007). Boquel et al. (2013) performed RT-PCR to identify PVY in the stylet to determine the effect of mineral oil on the viral acquisition and found a reduction in virus in the stylet of treated aphids. Kim et al. (2016) used an RT-PCR aided boiling procedure to find PVY in the complete body of a single aphid. RNAi has been utilized as a potent laboratory tool to investigate biological processes and gene functioning in many organisms. Furthermore, it is a potential tool in both the medical and agricultural fields for gene therapy and insect control, respectively (Agrawal et al., 2003; Huvenne and Smagghe, 2010; Zhu, 2013; Setten et al., 2019; Wesley and Luciano, 2019). Some viruses have found mechanisms to defeat the plant RNAi defense system as a result of an evolutionary relationship between host plant RNAi and target viruses. The multipurpose HC-Pro, for example, which is implicated in aphid transmission of potyviruses, may also decrease plant RNAi defense mechanisms (Lewsey et al., 2009; Rodamilans et al., 2018; Pollari et al., 2020).

## Vector control strategies

Pesticides alter vector behavior, which can result in reduced or greater PVY transmission rates through increased death, decreased probing, or increased flight and movement. Pesticides and antagonists that can diminish the prevalence of persistently transmitted PLRV are ineffective over non-persistently transmitted PVY since they do not kill the vectors quickly to avoid transmission (Shanks and Chapman, 1965; Boquel et al., 2015). Straw mulch considerably decreased the incidence of PVY in descendent tubers, with reductions extending from 50 to 70% in all 3 years. Straw mulching may affect aphid sight and lowers the distinction between vegetation background canopy (Doring and Schmidt, 2007; Döring, 2014). The use of mineral oil shortened the period of PVY retention in the stylet of *M. persicae* from 17 h to only 2 min (Wróbel, 2009).

**Figure 5:**
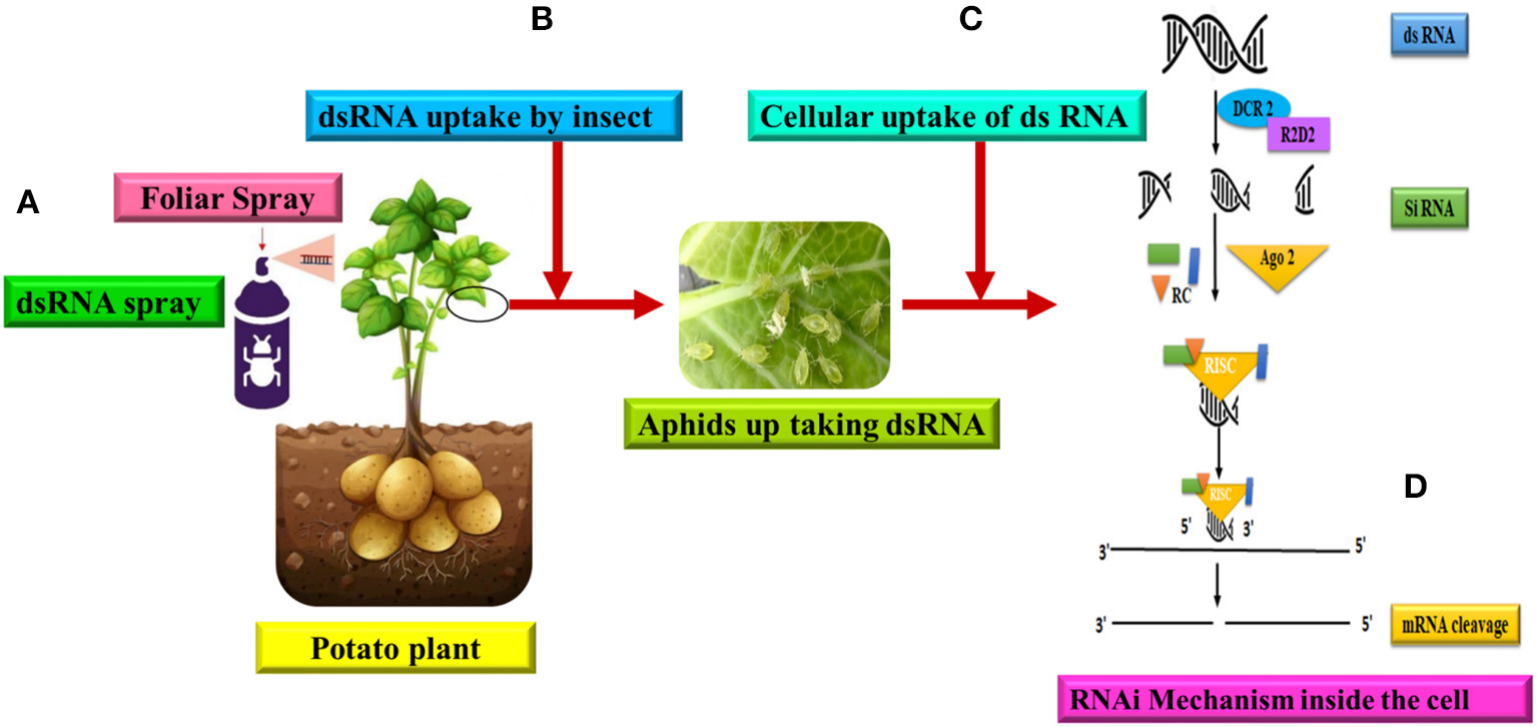
RNAi mediated gene silencing and direct dsRNA uptake for management of PVY. **(A)** Foliar spray of artificial exogenous dsRNA to the potato plant, **(B)** Piercing-sucking stylets acquire the virus from the phloem tissues of the plant, **(C)** Uptake of dsRNA to the cells of aphid, **(D)** The cellular RNAi mechanism of gene silencing in insects is illustrated. The RNAi molecular mechanism began in the cell with the Dicer 2 (Dcr2) enzyme cleaving dsRNA into short 21-24 nucleotide small interfering RNA (siRNA) duplexes. Following that, siRNAs are bound by Argonaute 2 (Ago2) proteins, which assemble the siRNA duplexes into the RNA-induced silencing complex (RISC), a multiprotein-siRNA structure. The RISC then mediates the cleavage of mRNA transcripts complementary to the integrated guide strand, thereby silencing the target gene and blocking protein translation (Modified from Cooper et al., 2019).

RNAi is a molecular technique that targets gene expression in organisms by producing and delivering dsRNA (Figure 5) (Aalto et al., 2007; Carthew and Sontheimer, 2009; Lam et al., 2015; Kanakala and Ghanim, 2016). The RNAi pathway is activated by the introduction of dsRNA (Fang et al., 2020). When dsRNA strands are recognized in a cell, they are cleaved into small interfering RNA (siRNA, 19–24 nucleotides) by the RNase III ribonuclease Dicer. The siRNAs subsequently form the RNAi-induced silencing complex RISC by interacting with an enzyme system (containing Argonaute proteins). RISC undergoes conformational modifications, allowing the lead strand of the siRNA inside the complex to identify the base and pair with the corresponding area on the targeted mRNA sequence. This complementary sequence-specific base pairing results in the breakdown or inhibition of the targeted mRNA's translation. RNAi has been used to discover receptors of NCNP aphid-transmitted plant viruses such as potyviruses, cucumoviruses, and Caulimovirus (Liu et al., 2015; Liang and Gao, 2017; Webster et al., 2017, 2018; Deshoux et al., 2018, 2020). RNAi-based suppression of chaperone-usher pathway (Cup) genes has been used for pest management (Shang et al., 2020) as well as physiological research, such as metamorphosis (Jan et al., 2017). RNAi has also been used to limit plant viral transmission by suppressing virus replication in its vector (Fang et al., 2020). In RNAi research, dsRNA delivery techniques including microinjection, feeding, and soaking have all been utilized to assure dsRNA delivery to insects. It has been established that improved administration by feeding using artificial nanoparticles to safeguard dsRNA or modified microorganisms that create dsRNA improves the efficiency of RNAi in insects (Yu et al., 2013; Kolliopoulou et al., 2017; Christiaens et al., 2020). Some of the shortcomings of the RNAi technique can be overcome with the availability of modern genome editing technologies such as CRISPR-Cas (Clustered Regularly Interspaced Short Palindromic Repeats-Cas). Instead of using temporary mRNA knockdown to explore gene functions, they might be entirely knocked out using genome editing (Cooper et al., 2019; Le Trionnaire et al., 2019). Aphid genome editing methodology was recently disclosed (Le Trionnaire et al., 2019; Tyagi et al., 2020). This is a fantastic tool that will tremendously aid research into the interactions between aphids and viruses, among other things.

Apart from RNAi (Pooggin, 2017), recent advancements in CRISPR-Cas system-mediated DNA or RNA editing/interference in plants enabled them as key components of plant protection (Cao et al., 2020). CRISPR-Cas13 was used to confer broad spectrum resistance by Zhan et al. (2019) to protect potato plants from PVY by designing sgRNAs targeting P3 (a membrane protein involved in pathogenicity, movement, systemic infection, and virus replication), CI (which forms the laminate cytoplasmic inclusion bodies, involved in infection and virus movement), NIB (an RNA-dependent RNA polymerase (RdRp) that participates in the replication of the viral RNA) and CP (which is active in virion assembly, cell-to-cell and systemic movement, and vector transmission) encoding regions of PVY RNA. The transgenic potato lines with the greatest Cas13a-sgRNA expression levels were chosen for the PVY challenge. Transgenic potato plants expressing P3, CI, NIB, or CP expressed Cas13a-sgRNA with more than 99% less virus accumulation than WT (non-transformed control plants). WT potato plants at 25 dpi showed typical PVY mosaic symptoms, whereas the edited plants exhibited no disease symptoms. Transgenic potato Cas13a/sgRNA expression level directly correlated with viral resistance. The transgenic plants also faced other PVY strains (PVYN and recombinant PVYN:O) that expressed CP-sgRNA. In transgenic plants, virus titers were reduced by more than 90%. In addition, it was tested whether the PVY-resistant transgenic plants had resistance to other non-related potyviruses such as *Potato Virus A* and *Potato Virus S* that share little in common with PVY. The spacer sequences of PVA and PVS were < 35% similar to their respective targets when compared with their respective sgRNA sequences. As far as virus accumulation is concerned, there was no significant difference between wild type potato plants and transgenic potatoes expressing CP-sgRNA. Further, the interference performance in transgenic lines with maximum and minimum expression profiles were compared and PVY inhibition was found to be positively correlated with the LshCas13a/sgRNA transcriptional dynamics. A sgRNA designed against a conserved region was found to confer broad-spectrum viral resistance. Meanwhile, introducing the CRISPR-Cas13a into potato plants was found to confer high-level PVY resistance. Moreover, CRISPR-Cas9 technology targeting mutation of the coilin gene (Makhotenko et al., 2019) created PVY resistance. Over expression of the eukaryotic translation initiation factor, eIF4E enabled potato resistance to PVY (Gutierrez Sanchez et al., 2020) by modifying the viral replication process. Further, noble possible targets for antiviral strategies like plant translation factors (eIF4A-like helicases, eIF3, eEF1A, and eEF1B) that specifically interact with viral RNAs and proteins and regulate various aspects of the infection cycle. Many naturally occurring plant recessive resistance genes have been mapped to mutate in isoforms of translation initiation factors eIF4E and eIF4G. The involvement of eIF4E in imparting natural resistance through genes like *pvr1/pvr12* and *pot-1* in *Capsicum spp* (point mutations) and *Solanum habrochaites* plants, respectively, was also documented (Sanfaçon, 2015).

## Building resistance against PVY: Breeding tools and strategies

Developing disease-resistant crops and reducing agricultural losses due to pathogens require an understanding of the plant immune system. In potato breeding, there are so many desirable features to look for in a cultivar. Breeders have the challenge of combining high yield and acceptable quality features with resilience to a wide range of diseases and pests in different countries. Along with this, the autotetraploid nature of *Solanum tuberosum* adds extra difficulty to the breeding procedure (Solomon-Blackburn and Barker, 2001). This challenge has been addressed, in part, by creating multiplex resistant parents that carry 3–4 copies of the dominant resistance gene (triplex/quadruplex) (Bradshaw and Mackay, 1994). As long as these resistant parents cross with a susceptible one, the offspring will be resistant as well. PVY genetic resistance is now derived from three distinct sources, each of which confers severe resistance to a diverse range of PVY strains (Valkonen, 1994; Chikh-Ali et al., 2020). These are derived from *S. stoloniferum* (Rysto) (Ross, 1958), *S. tuberosum* ssp. *andigena* (Ryadg) (Munoz et al., 1975), and *S. chacoense* (Rychc) (Asama, 1982). Three genes (Ryadg, Rysto, and Rychc) derived from wild species and inserted into a cultivated variety offer extreme resistance to PVY. Rychc is only reported to be found in a small number of varieties in North America, while Rysto and Ryadg are both frequently employed in breeding efforts around the world (Elison et al., 2021).

PVY had a novel source of genetic resistance from three diploid biparental potato populations. Using a developmental and reproductive toxicology (DaRT) study of the 08H1 cross, the 05H1 and 08H1 populations were found to be resistant to PVY due to the presence of a single dominant gene located on chromosome 9 (Torrance et al., 2020). The majority of plant resistance genes encode NLR receptors, which bind nucleotides intracellularly. TIR-NLRs are genes that have an N-terminal TIR domain, while CCNLRs contain an N-terminal coiled coil domain (Jones et al., 2016). For the detection and assembly of full-length genes encoding NLR that co-segregate with resistance traits, Pacific Biosciences' single-molecule real-time sequencing (SMRT RenSeq) (Witek et al., 2016) is a valuable technique. When it comes to monitoring NLR sequences, SMRT RenSeq technology (Grech-Baran et al., 2020) is more reliable and less expensive than whole-genome sequencing (Novy et al., 2017; Slater et al., 2020). Improvements in marker technologies, especially the marker-assisted selection and bulked segregant analysis (BSA) can be used (Michelmore et al., 1991). The molecular marker related to Rychc was found in five russet breeding clones (Elison et al., 2021; Ross et al., 2021). The PVY-resistant cultivars Konafubuki (Hosaka et al., 2001), Harimaru (Fujimatsu et al., 2018), and the breeding line Saikai 35 have all been developed by Japanese breeders using Rychc (Mori et al., 2012). Identifying existing populations in search of novel genetic markers is anticipated to be extremely beneficial as more and more valuable features are discovered and genetic markers are created. Transgenic resistance can be produced utilizing non-pathogen-derived sequences using a variety of other methods. Non-specific (wide-spectrum) resistance is claimed to be generated by several of them, which may offer significant advantages if paired with more specific transgenic resistance (Solomon-Blackburn and Barker, 2001). One usage of host or pathogen-derived transgenes is to insert genes that are lacking from an existing cultivar. NewLeaf^®^ series of cultivars are based on known cultivars like as “Russet Burbank” and Shepody, which have been reworked for “NatureMark.” Pesticide resistant insects (Colorado beetle) and disease resistant pathogens (PLRV and PVY) can be found in their genomes (Monsanto, 1997). Crop breeding efforts around the world are increasingly focusing on developing crops that are resistant to disease. There are numerous issues facing global agriculture, such as climate change and an increasing human population, which necessitate a better understanding of plant defense responses to viruses.

## Conclusion

The PVY transmission dynamics are complex and reported to be governed by a multitude of factors and their associated interactions. There is much work to be done to enable better understanding of all the mechanisms at a molecular, biochemical, and bioinformatics level. PVY has been reported to have both horizontal as well as vertical transmission modes (Hegde et al., 2021) by different aphid species in a non-circulative and non-persistence manner with differential transmission efficiency (Lacomme et al., 2017). Diverse vector management strategies were adopted worldwide, especially through chemical alternatives, yet the development of resistance in aphid populations and indiscriminate pesticide usage has had severely detrimental effects on the environment as well as on beneficial organisms. This necessitates a suitable understanding of the inter-relationship between potato, aphid, and PVY that can be further exploited to manage the virus-vector complex. Although molecular based techniques have been identified and are in use (Glais et al., 2017), molecular responses of PVY in plants, Gene Ontology (GO) analysis, and bioinformatics of host plant-aphid-PVY transmissions can open up new ways to culminate the vector as well as the vector-mediated virus transmission. Furthermore, aphid mediated PVY transmissions illustrated stronger genetic drawbacks than mechanical modes of transmission. In this regard, virus population, viral-host association, intra-host movement of the virus, and within-plant diversity of virus populations can be the focus of research that will further provide quantitative and qualitative evidence for understanding the complex transmissions. Differential breeding approaches can be adopted additionally, which will help develop resistant/tolerant cultivars, and thus can help in the adoption of more environmentally friendly management strategies.

## Future prospective

Viral transmission studies are a well-established approach to investigating the control of diseases. They become an area of research prioritization in recent years due to higher mutation rates among viruses. The following considerations should be the prime objectives in the current scenario:

1. Adopting a suitable diagnostic tool for proper identification (Glais et al., 2017) can be useful in understanding PVY and their characteristic mode of transmission (by various strains), genome organization and protein function, genetic diversity, molecular and bioinformatics aspects of PVY transmission by aphids, methods used in detection and characterization of PVY (Medina Cárdenas et al., 2017), transcriptomics level of response, identification, and monitoring of virus transmission, and vector control strategies in an exclusive manner.
2. Farmers should focus efforts on detecting and monitoring diseased plants and removing virus infected plants from the field. Nevertheless, depending on the cultivar, virus diseased plants can be missed during visual observations, in particular in the early stages of cultivation. Therefore, there is a need for fast and objective disease detection. Disease symptoms can be detected with machine vision techniques using hyperspectral cameras. Early detection of diseased plants with modern vision techniques can significantly reduce costs. Laboratory experiments in the past showed that hyperspectral imaging clearly could distinguish healthy from PVY infected potato plants. A hyperspectral line-scan camera can be used and a convolutional neural network could be adapted for hyperspectral images (Polder et al., 2019).
3. Growing potato cultivars resistant to PVY offers the easiest and most cost-effective solution to preventing the losses caused by PVY. In search of broad-spectrum resistance to PVY, the marker-assisted selection provides an efficient approach for the selection of traits governed by major genes or quantitative trait loci (QTLs) with large effects. Thus, resistance breeding approaches can be followed to adopt an eco-friendly management strategy.
4. Regarding genetically engineered resistance based on RNA silencing, the basal antiviral defense system of plants along with CRISPR-Cas13a can provide protection to potatoes against PVY (Nasr-Eldin et al., 2019). Although various methods were used to silence RNA molecules through artificial feeding or injection, expressing virus siRNA molecules in plant hosts for insect feeding can be the best approach for developing RNAi-based pest control methods. CRISPR-Cas9 targeted mutation of the coilin gene, overexpression of eIF4E, and expression of LshCas13a/sgRNAs can further be helpful in this approach to impart resistance/tolerance to PVY. The use of silencing genes will be effective in controlling persistent viruses, since the genes involved in transmission will be silenced, and the vector itself can be controlled as well, allowing control of not only persistent viruses but also non-persistent and semi-persistent ones.
5. Recently, new breeding techniques have been utilized to knock-out potato genes/factors like eukaryotic translation initiation factors [elF4E and isoform elF(iso)4E)], that interact with viruses to assist viral infection, and vacuolar invertase, a core enzyme in CIS (Miroshnichenko et al., 2020). In this context, CRISPR technology is predicted to reduce the cost of potato production and is likely to pass through the regulatory process being marker- and transgene-free (Hameed et al., 2020). Under new breeding techniques, Pathogen-Derived Resistance (PDR) can be obtained by targeting coat protein (CP) regions using chimeric CP, RNA silencing, and Hp RNAi (Hairpin RNA interference), which can provide higher resistance to PVY. The HCpro region can be targeted through Hp-RNAi, which will reduce the virus titer (Hameed et al., 2020). Further host-gene mediated PVY resistance can be obtained through targeting the Y-1 gene, eIF4E-1 allele, and Nz gene to achieve systemic cell death and resistance against PVY (Vidal et al., 2002; Cavatorta et al., 2011; Chikh-Ali et al., 2014). The major limiting factors in the target selection in RNAi are the possible risks of off-site targeting in RNAi, loss of transgene expression (integrations in the untranslated genomic region, introns), and the presence of natural polymorphism. RNAi-mediated gene silencing provides selective but non-absolute specificity. Off-site targeting risks can arise when transgene-derived siRNAs silence host genes due to sufficient nucleotide sequence complementarity (Casacuberta et al., 2015). Further, off-target silencing might result in unanticipated phenotypic alterations along with linkage drag that have a considerable influence on agronomic performance or have other negative impacts on host genes (Jackson and Linsley, 2010). In this context, various bioinformatic tools have been developed to overcome shortcomings that can computationally predict efficient sgRNA targeting sites avoiding any offsite targeting. For precise targeting, some web-based tools like CRISPR Design, Cas-OFFinder, CCTop, CHOPCHOP, E-CRISP, CROP-IT, CRISPR-P V2.0, CRISPRPLANT, and SYNTHEGO can be utilized (Hameed et al., 2020).
6. Altering host plant-insect vector-virus interaction is the most effective way to achieve potential success in PVY control. Physio-morphological changes were reported as a result of the virus spreading to nearby cells that lack active defense systems. Although a variety of passive and active defense systems against plant pathogens were detected earlier, physio-chemical and molecular analyses of the plant-pathogen interaction are still in infancy (Whitham et al., 2006; Nasr-Eldin et al., 2018). Altered plant-pathogen interaction can modify viral replication, which ultimately induces diverse pathogenesis-dependent defensive reactions (O'Donnell et al., 2003). Moreover, plants' natural defense mechanisms can be artificially induced using biotic or biotic elicitors, and thus viral diseases can be controlled, promoting systemic acquired resistance (SAR) (Falcioni et al., 2014).

Thus, the current review emphasizes a holistic approach involving the adoption of suitable identification, resistance breeding approaches including new breeding techniques, identifying the active defensive pathways and aphid elicitors involved in pathogen transmission, which will ultimately be helpful for solanaceous growers.

## Author contributions

TB, IS, DM, JK, PM, and AP: conceptualization, writing—original draft preparation, preparation of tables, and supervision. DM, VS, MN, MAA, PB, and MA: preparation of figures, supervision, review, and editing. All authors have read and approved the manuscript.

## Conflict of interest

The authors declare that the research was conducted in the absence of any commercial or financial relationships that could be construed as a potential conflict of interest.

## Publisher's note

All claims expressed in this article are solely those of the authors and do not necessarily represent those of their affiliated organizations, or those of the publisher, the editors and the reviewers. Any product that may be evaluated in this article, or claim that may be made by its manufacturer, is not guaranteed or endorsed by the publisher.

## References

[B1] Aalto A. P. Sarin L. P. van Dijk A. A. Saarma M. Poranen M. M. Arumäe U.. (2007). Large-scale production of dsRNA and siRNA pools for RNA interference utilizing bacteriophage phi6 RNAdependent RNA polymerase. RNA 13, 422–429. 10.1261/rna.348307 17237359 PMC1800515

[B2] Aarts N. Metz M. Holub E. Staskawicz B. J. Daniels M. J. Parker J. E. (1998). Different requirements for EDS1 and NDR1 by disease resistance genes define at least two R gene-mediated signaling pathways in Arabidopsis. Proc. Natl. Acad. Sci. U. S. A. 95, 10306–10311. 10.1073/pnas.95.17.10306 9707643 PMC21504

[B3] Adams M. J. Antoniw J. F. Beaudoin F. (2005). Overview and analysis of the polyprotein cleavage sites in the family Potyviridae. Mol. Plant. Pathol. 6, 471–487. 10.1111/j.1364-3703.2005.00296.x 20565672

[B4] Agrawal N. V. N. Dasaradhi P. V. Mohmmed A. Malhotra P. K. Bhatnagar R. K. Mukherjee S. K. (2003). RNA interference: biology, mechanism, and applications. Microbiol. Mol. Biol. Rev. 67, 657–685. 10.1128/MMBR.67.4.657-685.2003 14665679 PMC309050

[B5] Aleman-Verdaguer M. E. Goudou-Urbino C. Dubern J. B. Beachy R. N. C. Fauquet C. (1997). Analysis of the sequence diversity of the P1, HC, P3, NIb and CP genomic regions of several yam mosaic Potyvirus isolates: implications for the intraspecies molecular diversity of potyviruses. J. Gen. Virol. 78, 1253–1264. 10.1099/0022-1317-78-6-1253 9191916

[B6] Ammar E. D. Jarlfors U. Pirone T. (1994). Association of Potyvirus Helper component protein with virions and the cuticle lining the maxillary food canal and foregut of an aphid vector. Phytopathology. 84, 1054–1060. 10.1094/Phyto-84-1054

[B7] Anandalakshmi R. Pruss G. J. Ge X. Marathe R. Mallory A. C. Smith T. H.. (1998). A viral suppressor of gene silencing in plants. Proc. Natl. Acad. Sci. U. S. A. 95, 13079–13084. 10.1073/pnas.95.22.13079 9789044 PMC23715

[B8] Asama K. (1982). New potato variety “Konafubuki”. Boll. Hokkaido Pref. Agric. Exp. Stn. 48, 75–84.

[B9] Baebler Š. Coll A. Gruden K. (2020). Plant molecular responses to potato virus Y: a continuum of outcomes from sensitivity and tolerance to resistance. Viruses 12, 217. 10.3390/v12020217 32075268 PMC7077201

[B10] Baebler Š. Witek K. Petek M. Stare K. Tušek-Znidaric M. Pompe-Novak M.. (2014). Salicylic acid is an indispensable component of the Ny-1 resistance-gene-mediated response against potato virus Y infection in potato. J. Exp. Bot. 65, 1095–1109. 10.1093/jxb/ert447 24420577 PMC3935562

[B11] Balint-Kurti P. (2019). The plant hypersensitive response: concepts, control and consequences. Mol. Plant Pathol. 20, 1163–1178. 10.1111/mpp.12821 31305008 PMC6640183

[B12] Betancourt M. Fereres A. Fraile A. García-Arenal F. (2008). Estimation of the effective number of founders that initiate an infection after aphid transmission of a multipartite plant virus. J. Virol. 82, 12416–12421. 10.1128/JVI.01542-08 18842732 PMC2593348

[B13] Blanc S. López-Moya J. J. Wang R. García-Lampasona S. Thornbury D. W. Pirone T. P. (1997). A specific interaction between coat protein and helper component correlates with aphid transmission of a Potyvirus. Virology 231, 141–147. 10.1006/viro.1997.8521 9143313

[B14] Boiteau G. Singh R. P. Parry R. H. Pelletier Y. (1988). The spread of PVY in New Brunswick potato fields: timing and vectors. Am. Potato. J. 65, 639–649. 10.1007/BF02854832

[B15] Bond J. K. (2014). “Potato utilization and markets,” in The Potato: Botany, Production and Uses (Wallingford: CABI Publishing), 29–44. 10.1079/9781780642802.0029

[B16] Boquel S. Ameline A. Giordanengo P. (2011). Assessing aphids potato virus Y-transmission efficiency: a new approach. J. Virol. Methods. 178, 63–67. 10.1016/j.jviromet.2011.08.013 21884729

[B17] Boquel S. Giguère M. Clark C. Nanayakkara U. Zhang J. Pelletier Y. (2013). Effect of mineral oil on potato virus Y acquisition by *Rhopalosiphum padi*. Entomol. Exp. Appl. 148, 48–55. 10.1111/eea.12070

[B18] Boquel S. Zhang J. Goyer C. Giguère M. A. Clark C. Pelletier Y. (2015). Effect of insecticide-treated potato plants on aphid behavior and potato virus Y acquisition. Pest. Manag. Sci. 71, 1106–1112. 10.1002/ps.3892 25159012

[B19] Bosquee E. Boullis A. Bertaux M. Francis F. Verheggen F. J. (2018). Dispersion of *Myzus persicae* and transmission of potato virus Y under elevated CO_2_ atmosphere. Entomol. Exp. Appl. 166, 380–385. 10.1111/eea.12661

[B20] Bradshaw J. E. Mackay G. R. (eds.). (1994). “Breeding strategies for clonally propagated potatoes” in Potato Genetics, (Invergowrie: CA B International), 467–497. 10.1079/9780851988696.0000

[B21] Cao Y. Fanning S. Proos S. Jordan K. Srikumar S. (2017). A review on the applications of next generation sequencing technologies as applied to food-related microbiome studies. Front. Microbiol. 8, 1829. 10.3389/fmicb.2017.01829 29033905 PMC5627019

[B22] Cao Y. Zhou H. Zhou X. Li F. (2020). Control of plant viruses by CRISPR/Cas system-mediated adaptive immunity. Front Microbiol. 11, 593700. 10.3389/fmicb.2020.593700 33193268 PMC7649272

[B23] Carrington J. C. Dougherty W. G. (1987a). Small nuclear inclusion protein encoded by a plant Potyvirus genome is a protease. J Virol. 61, 2540–2548. 10.1128/JVI.61.8.2540-2548.1987 16789265 PMC255690

[B24] Carrington J. C. Dougherty W. G. (1987b). Processing of the tobacco etch virus 49K protease requires autoproteolysis. Virology 160, 355–362. 10.1016/0042-6822(87)90006-7 18644573

[B25] Carthew R. W. Sontheimer E. J. (2009). Origins and mechanisms of miRNAs and siRNAs. Cell 136, 642–655. 10.1016/j.cell.2009.01.035 19239886 PMC2675692

[B26] Casacuberta J. M. Devos Y. Du Jardin P. Ramon M. Vaucheret H. Nogué F. (2015). Biotechnological uses of RNAi in plants: Risk assessment considerations. Trends Biotechno 33, 145–147. 10.1016/j.tibtech.2014.12.003 25721261

[B27] Castel B. Ngou P. M. Cevik V. Redkar A. Kim D. S. Yang Y.. (2019). Diverse NLR immune receptors activate defence via the RPW8-NLR NRG1. New Phytol. 222, 966–980. 10.1111/nph.15659 30582759

[B28] Cavatorta J. Perez K. W. Gray S. M. Van Eck J. Yeam I. Jahn M. (2011). Engineering virus resistance using a modified potato gene. Plant. Biotech. J. 9, 1014–1021. 10.1111/j.1467-7652.2011.00622.x 21668622

[B29] Chikh-Ali M. Rowley J. S. Kuhl J. Gray S. M. Karasev A. V. (2014). Evidence of a monogenic nature of the Nz gene conferring resistance against *Potato virus Y* strain Z (PVY Z) in potato. Am. J. Potato Res. 91, 649–654. 10.1007/s12230-014-9395-7

[B30] Chikh-Ali M. Tran L. T. Price W. J. Karasev A. V. (2020). Effects of the age-related resistance to potato virus Y in potato on the systemic spread of the virus, incidence of the potato tuber necrotic ringspot disease, tuber yield, and translocation rates into progeny tubers. Plant Dis. 104, 269–275. 10.1094/PDIS-06-19-1201-RE 31746695

[B31] Christiaens O. Whyard S. Vélez A. M. Smagghe G. (2020). Double-stranded RNA technology to control insect pests: current status and challenges. Front. Plant. Sci. 11, 451. 10.3389/fpls.2020.00451 32373146 PMC7187958

[B32] Chung B. N. Canto T. Tenllado F. Choi K. S. Joa J. H. Ahn J. J.. (2016). The Effects of High temperature on Infection by potato virus Y, Potato virus A, and Potato leafroll virus. Plant. Pathol. J. 32, 321–328. 10.5423/PPJ.OA.12.2015.0259 27493607 PMC4968642

[B33] Clark M. F. Adams A. N. (1977). Characteristics of the microplate method of enzyme-linked immunosorbent assay for the detec- tion of plant viruses. J. Gen. Virol. 34, 475–483. 10.1099/0022-1317-34-3-475 323416

[B34] Cooper A. M. Silver K. Zhang J. Park Y. Zhu K. Y. (2019). Molecular mechanisms influencing efficiency of RNA interference in insects. Pest. Manag. Sci. 75, 18–28. 10.1002/ps.5126 29931761

[B35] Cooper J. I. Jones A. T. (1984). The use of terms for responses of plants to viruses: a reply to recent proposals. Phytopathology 73, 127–128. 10.1094/Phyto-73-127

[B36] Crosslin J. M. (2013). PVY: an old enemy and a continuing challenge. Am. J. Potato. Res. 90, 2–6. 10.1007/s12230-012-9286-8

[B37] Crosslin J. M. Hamm P. B. Shiel P. J. Hane D. C. Brown C. R. Berger P. H. (2005). Serological and molecular detection of tobacco veinal necrosis isolates of potato virus Y (PVYN) from potatoes grown in the western United States. Am. J. Potato. Res. 82, 263–269. 10.1007/BF02871955

[B38] Da Silva W. Kutnjak D. Xu Y. Xu Y. Giovannoni J. Elena S. F.. (2020). Transmission modes affect the population structure of potato virus Y in potato. PLoS Pathog. 16, e1008608. 10.1371/journal.ppat.1008608 32574227 PMC7347233

[B39] Dáder B. Fereres A. Moreno A. Trebicki P. (2016). Elevated CO2 impacts bell pepper growth with consequences to *Myzus persicae* life history, feeding behaviour and virus transmission ability. Sci. Rep. 6, 19120. 10.1038/srep19120 26743585 PMC4705479

[B40] Danecek P. Auton A. Abecasis G. Albers C. A. Banks E. DePristo M. A.. (2011). The variant call format and VCFtools. Bioinformatics 27, 2156–2158. 10.1093/bioinformatics/btr330 21653522 PMC3137218

[B41] Deepti N. LaTourrette K. Souza P. F. N. Garcia-Ruiz H. (2019). Genome-wide variation in potyviruses. Front. Plant. Sci. 10, 1439. 10.3389/fpls.2019.01439 31798606 PMC6863122

[B42] Deshoux M. Masson V. Arafah K. Voisin S. Guschinskaya N. Van Munster M.. (2020). Cuticular structure proteomics in the pea aphid *Acyrthosiphon pisum* reveals new plant virus receptor candidates at the tip of maxillary stylets. J. Proteome Res. 19, 1319–1337. 10.1021/acs.jproteome.9b00851 31991085 PMC7063574

[B43] Deshoux M. Monsion B. Uzest M. (2018). Insect cuticular proteins and their role in transmission of phytoviruses. Curr. Opin. Virol. 33, 137–143. 10.1016/j.coviro.2018.07.015 30245214 PMC6291435

[B44] Dietzgen R. G. Mann K. S. Johnson K. N. (2016). Plant virus–insect vector interactions: current and potential future research directions. Viruses 8, 303. 10.3390/v8110303 27834855 PMC5127017

[B45] Dijkstra J. Jager C. D. (1998). Practical Plant Virology: Protocols and Exercises. Heidelberg; Berlin: Springer Science+Business Media. 10.1007/978-3-642-72030-7

[B46] Dobnik D. Lazar A. Stare T. Gruden K. Vleeshouwers V. G. Žel J. (2016). *Solanum venturii*, a suitable model system for virus-induced gene silencing studies in potato reveals StMKK6 as an important player in plant immunity. Plant Metho. 12, 29. 10.1186/s13007-016-0129-3 27213007 PMC4875682

[B47] Dogimont C. Palloix A. Daubze A. M. Marchoux G. Selassie K. G. Pochard E. (1996). Genetic analysis of broad-spectrum resistance to potyviruses using doubled haploid lines of pepper (*Capsicum annuum* L.) Euphotic 88, 231–239.

[B48] Domingo E. Perales C. (2019). Viral quasispecies. PLoS Genet. 15, e1008271. 10.1371/journal.pgen.1008271 31622336 PMC6797082

[B49] Döring T. F. (2014). How aphids find their host plants, and how they don't. Ann. Appl. Biol. 165, 3–26. 10.1111/aab.12142

[B50] Doring T. F. Schmidt T. (2007). Response of apterous potato aphids to visual contrasts (Hemiptera: Aphididae). Entomol. Genet. 30, 190–191 10.1127/entom.gen/30/2007/190

[B51] Duffy S. (2018). Why are RNA virus mutation rates so damn high? PLoS Biol. 16, e3000003. 10.1371/journal.pbio.3000003 30102691 PMC6107253

[B52] Dupuis B. Bragard C. Schumpp O. (2019). Resistance of potato cultivars as a determinant factor of potato virus Y (PVY) epidemiology. Potato Res. 62, 123–138. 10.1007/s11540-018-9401-4

[B53] Elena S. F. Bedhomme S. Carrasco P. Cuevas J. M. De la Iglesia F. Lafforgue G.. (2011). The evolutionary genetics of emerging plant RNA viruses. Mol. Plant. Microbe. Interact. 24, 287–293. 10.1094/MPMI-09-10-0214 21294624

[B54] Elison G. L. Novy R. G. Whitworth J. L. (2021). Russet potato breeding clones with extreme resistance to potato virus Y conferred by Rychc as well as resistance to late blight and cold-induced sweetening. Am. J. Potato. Res. 98, 411–419. 10.1007/s12230-021-09852-1

[B55] Falcioni T. Ferrio J. P. Del Cueto A. I. Giné J. Achón M. Á. Medina V. (2014). Effect of salicylic acid treatment on tomato plant physiology and tolerance to potato virus X infection. Eu. J. Plant. Pathol. 138, 331–345. 10.1007/s10658-013-0333-1

[B56] Fang Y. Choi J. Y. Park D. H. Park M. G. Kim J. Y. Wang M.. (2020). Suppression of rice stripe virus replication in *Laodelphax striatellus* using vector insect-derived double stranded RNAs. Plant. Pathol. J. 36, 280–288. 10.5423/PPJ.OA.03.2020.0052 32547343 PMC7272848

[B57] Fereres A. Moreno A. (2009). Behavioural aspects influencing plant virus transmission by homopteran insects. Virus Res. 141, 158–168. 10.1016/j.virusres.2008.10.020 19152819

[B58] Fox A. Collins L. E. Macarthur R. Blackburn L. F. Northing P. (2017). New aphid vectors and efficiency of transmission of Potato virus A and strains of Potato virus Y in the UK. Plant. Pathol. 66, 325–335. 10.1111/ppa.12561

[B59] Fuentes S. Jones R. A. C. Matsuoka H. Ohshima K. Kreuze J. Gibbs A. J. (2019). Potato virus Y; the Andean connection. Virus. Evol. 5, vez037. 10.1093/ve/vez037 31559020 PMC6755682

[B60] Fujimatsu M. Hashizume H. Fudan T. Koma Y. Sanetomo R. Ono S.. (2018). Harimaru: a new potato variety for a local specialty. Breed. Sci. 68, 284–288. 10.1270/jsbbs.17109 29875613 PMC5982187

[B61] Funke C. N. Nikolaeva O. V. Green K. J. Tran L. T. Chikh-Ali M. Quintero-Ferrer A.. (2017). Strain-specific resistance to potato virus Y (PVY) in potato and its effect on the relative abundance of PVY strains in commercial potato fields. Plant. Dis. 101, 20–28. 10.1094/PDIS-06-16-0901-RE 30682299

[B62] Gadhave K. R. Gautam S. Rasmussen D. A. Srinivasan R. (2020). Aphid transmission of Potyvirus: the largest plant-infecting RNA virus genus. Viruses 12, 773. 10.3390/v12070773 32708998 PMC7411817

[B63] Glais L. Bellstedt D. U. Lacomme C. (2017). “Diversity, characterisation and classification of PVY,” in Potato Virus Y: Biodiversity, Pathogenicity, Epidemiology and Management, eds C. Lacomme, L. Glais, D. U. Bellstedt, B. Dupuis, A. V. Karasev, E. Jacquot, et al. (Le Rehu: Springer). 10.1007/978-3-319-58860-5_3

[B64] Gray S. De Boer S. Lorenzen J. Karasev A. Whitworth J. Nolte P.. (2010). Potato virus Y: an evolving concern for potato crops in the United States and Canada. Plant. Dis. 94, 1384–1397. 10.1094/PDIS-02-10-0124 30743397

[B65] Grech-Baran M. Witek K. Szajko K. Witek A. Morgiewicz K. Wasilewicz-Flis I.. (2019). Extreme resistance to Potato virus Y in potato carrying the Rysto gene is mediated by a TIR-NLR immune receptor. bioRxiv. [Preprint]. 10.1101/445031 31397954 PMC7004898

[B66] Grech-Baran M. Witek K. Szajko K. Witek A. I. Morgiewicz K. Wasilewicz-Flis I.. (2020). Extreme resistance to potato virus Y in potato carrying the Rysto gene is mediated by a TIR-NLR immune receptor. Plant. Biotechnol. J. 18, 655–667. 10.1111/pbi.13230 31397954 PMC7004898

[B67] Guo Y. Jia M. A. Yang Y. Zhan L. Cheng X. Cai J.. (2017). Integrated analysis of tobacco miRNA and mRNA expression profiles under PVY infection provids insight into tobacco-PVY interactions. Sci. Rep. 7, 4895. 10.1038/s41598-017-05155-w 28687775 PMC5501784

[B68] Gutierrez Sanchez P. A. Babujee L. Jaramillo Mesa H. Arcibal E. Gannon M. Halterman D.. (2020). Overexpression of a modified eIF4E regulates potato virus Y resistance at the transcriptional level in potato. BMC Genomics, 21, 18. 10.1186/s12864-019-6423-5 31906869 PMC6945410

[B69] Hameed A. Mehmood M. A. Shahid M. Fatma S. Khan A. Ali S. (2020). Prospects for potato genome editing to engineer resistance against viruses and cold-induced sweetening. GM Crops Food 11, 185–205. 10.1080/21645698.2019.1631115 31280681 PMC7518746

[B70] Handayani T. Gilani S. A. Watanabe K. N. (2019). Climatic changes and potatoes: how can we cope with the abiotic stresses? Breed. Sci. 69, 545–563. 10.1270/jsbbs.19070 31988619 PMC6977456

[B71] Harahagazwe D. Andrade-Piedra J. Parker M. Schulte-Geldermann E. (2018). Current situation of Rapid Multiplication Techniques for Early Generation Seed Potato Production in Sub-Saharan Africa. International Potato Center, CGIAR Research Program on Roots, Tubers and bananas (RTB). RTB Working Paper no*. 2* 018–1.

[B72] Hasiow-Jaroszewska B. Minicka J. Stachecka J. Borodynko N. Piekna-Paterczyk D. Pospieszny H. (2014). Diversity of the Polish isolates of potato virus Y (PVY) from tomato. Prog. Plant. Prot. 54, 288–292. 10.14199/ppp-2014-046

[B73] Hegde K. Kalleshwaraswamy C. M. Venkataravanappa V. (2021). Role of virus infection in seed tubers, secondary spread and insecticidal spray on the yield of potato in Deccan Plateau, India. Potato Res. 64, 339–351. 10.1007/s11540-020-09480-y

[B74] Hillung J. Elena S. Cuevas J. (2013). Intra-specific variability and biological relevance of P3N-PIPO protein length in potyviruses. BMC Evol. Biol. 13, 249. 10.1186/1471-2148-13-249 24225158 PMC3840659

[B75] Hosaka K. Hosaka Y. Mori M. Maida T. Matsunaga H. (2001). Detection of a simplex RAPD marker linked to resistance to potato virus Y in a tetraploid potato. Am. J. Potato. Res. 78, 191–196. 10.1007/BF02883544

[B76] Hu X. Nie X. He C. Xiong X. (2011). Differential pathogenicity of two different recombinant PVYNTN isolates in *Physalis floridana* is likely determined by the coat protein gene. Virol J. 8, 207. 10.1186/1743-422X-8-207 21548970 PMC3112444

[B77] Huet H. Gal-On A. Meir E. Lecoq H. Raccah B. (1994). Mutations in the helper component protease gene of zucchini yellow mosaic virus affect its ability to mediate aphid transmissibility. J. Gen. Virol. 75, 1407–1414. 10.1099/0022-1317-75-6-1407 8207404

[B78] Huson D. H. (1998). SplitsTree: analyzing and visualizing evolutionary data. Bioinformatics 14, 68–73. 10.1093/bioinformatics/14.1.68 9520503

[B79] Hutton F. Spink J. H. Griffin D. Kildea S. Bonner D. Doherty G.. (2015). Distribution and incidence of viruses in Irish seed potato crops. Ir. J. Agric. Food Res. 54, 98–106. 10.1515/ijafr-2015-001119271342

[B80] Huvenne H. Smagghe G. (2010). Mechanisms of dsRNA uptake in insects and potential of RNAi for pest control: a review. J. Insect. Physio. 56, 227–235. 10.1016/j.jinsphys.2009.10.004 19837076

[B81] ICTV Report on Virus Classification Taxon Nomenclature (2020). Positive-sense RNA viruses > *Potyviridae*. Genus Potyvirus. Available online at: https://talk.ictvonline.org/ictv-reports/ictv_online_report/positive-senserna-viruses/w/potyviridae/572/genus-potyvirus (accessed November 13 2020).

[B82] Jackson A. L. Linsley P. S. (2010). Recognizing and avoiding siRNA off-target effects for target identification and therapeutic application. Nat. Rev. Drug Discov. 9, 57–67. 10.1038/nrd3010 20043028

[B83] Jailani A. A. K. Shilpi S. Mandal B. (2017). Rapid demonstration of infectivity of a hybrid strain of potato virus Y occurring in India through overlapping extension PCR. Physiol. Mol. Plant. Pathol. 98, 62–68. 10.1016/j.pmpp.2017.03.001

[B84] Jan S. Liu S. Hafeez M. Zhang X. Dawar F. U. Guo J.. (2017). Isolation and functional identification of three cuticle protein genes during metamorphosis of the beet armyworm, *Spodoptera exigua*. Sci. Rep. 7, 16061. 10.1038/s41598-017-16435-w 29167522 PMC5700046

[B85] Janzac B. Willemsen A. Cuevas J. M. Glais L. Tribodet M. Verrier J. -L.. (2015). Brazilian potato virus Y isolates identified as members of a new clade facilitate the reconstruction of evolutionary traits within this species. Plant Pathol. 64, 799–807. 10.1111/ppa.12318

[B86] Jimenez J. Webster C. G. Moreno A. Almeida R. P. P. Blanc S. Fereres A.. (2017). Fasting alters aphid probing behaviour but does not universally increase the transmission rate of non-circulative viruses. J. Gen. Virol. 98, 3111–3121. 10.1099/jgv.0.000971 29134940

[B87] Jones J. D. G. Vance R. E. Dangl J. L. (2016). Intracellular innate immune surveillance devices in plants and animals. Science 354, aaf6395. 10.1126/science.aaf6395 27934708

[B88] Kaliciak A. Syller J. (2009). New hosts of potato virus Y (PVY) among common wild plants in Europe. Eur. J. Plant. Pathol. 124, 707–713. 10.1007/s10658-009-9452-0

[B89] Kanakala S. Ghanim M. (2016). RNA interference in insect vectors for plant viruses. Viruses. 8, 329. 10.3390/v8120329 27973446 PMC5192390

[B90] Karasev A. V. Gray S. M. (2013). Continuous and emerging challenges of potato virus Y in potato. Annu Rev Phytopathol. 51, 571–586. 10.1146/annurev-phyto-082712-102332 23915135

[B91] Kerlan C. Moury B. (2008). “Potato virus Y,” in Encyclopaedia of virology, eds B. W. J. Mahy and M. H. V. Regenmortel (Academic Press), p. 287?296.

[B92] Kezar A. Kavčič L. Polák M. Nováček J. Gutiérrez-Aguirre I. Žnidarič M. T.. (2019). Structural basis for the multitasking nature of the potato virus Y coat protein. Sci adv. 5, eaaw3808. 10.1126/sciadv.aaw3808 31328164 PMC6636993

[B93] Kim J. Cha D. J. Kwon M. Maharjan R. (2016). Potato virus Y (PVY) detection in a single aphid by onestep RT-PCR with boiling technique. Entomol. Res. 46, 278–285. 10.1111/1748-5967.12170

[B94] Kim S. B. Kang W. H. Huy H. N. Yeom S. I. An J. T. Kim S.. (2017). Divergent evolution of multiple virus-resistance genes from a progenitor in *Capsicum* spp. New Phytol. 213, 886–899. 10.1111/nph.14177 27612097

[B95] Kogovšek P. Pompe-Novak M. Baebler Š. Rotter A. Gow L. Gruden K.. (2010). Aggressive and mild potato virus Y isolates trigger different specific responses in susceptible potato plants. Plant. Pathol. 59, 1121–1132. 10.1111/j.1365-3059.2010.02340.x

[B96] Kolliopoulou A. Taning C. N. T. Smagghe G. Swevers L. (2017). Viral delivery of dsRNA for control of insect agricultural pests and vectors of human disease: prospects and challenges. Front. Physiol. 8, 399. 10.3389/fphys.2017.00399 28659820 PMC5469917

[B97] Kreuze J. F. Souza-Dias J. A. C. Jeevalatha A. Figueira A. R. Valkonen J. P. T. Jones R. A. C. (2020). “Viral diseases in potato,” in The Potato Crop: Its Agricultural, Nutritional And Social Contribution to Humankind, eds H. Campos and O. Ortiz (Peru: Springer), 389–430. 10.1007/978-3-030-28683-5_11

[B98] Kutnjak D. Rupar M. Gutierrez-Aguirre I. Curk T. Kreuze J. F. Ravnikar M. (2015). Deep sequencing of virus-derived small interfering RNAs and RNA from viral particles shows highly similar mutational landscapes of a plant virus population. J. Virol. 89, 4760–4769. 10.1128/JVI.03685-14 25673712 PMC4403455

[B99] Lacomme C. Pickup J. Fox A. Glais L. Dupuis B. Steinger T.. (2017). “Transmission and epidemiology of potato virus Y,” in Potato Virus Y Biodiversity, Pathogenicity, Epidemiology and Management (Edinburgh: Springer International Publishing), 141–176. 10.1007/978-3-319-58860-5_6

[B100] Lam J. K. Chow M. Y. Zhang Y. Leung S. W. (2015). siRNA versus miRNA as Therapeutics for Gene Silencing. Mol. Ther. Nucleic. Acids 4, e252. 10.1038/mtna.2015.23 26372022 PMC4877448

[B101] Lazar A. Coll A. Dobnik D. Baebler S. Bedina-Zavec A. Žel J.. (2014). Involvement of Potato (*Solanum tuberosum* L.) MKK6 in Response to potato virus Y. PLoS ONE 9, e104553. 10.1371/journal.pone.0104553 25111695 PMC4128675

[B102] Le Trionnaire G. Tanguy S. Hudaverdian S. Gleonnec F. Richard G. Cayrol B.. (2019). An integrated protocol for targeted mutagenesis with CRISPR-cas9 system in the pea aphid. Insect. Biochem. Mol. Biol. 110, 34–44. 10.1016/j.ibmb.2019.04.016 31015023

[B103] Lefort V. Longueville J. E. Gascuel O. (2017). SMS: Smart model selection in PhyML. Mol Biol Evol. 34, 2422–2424. 10.1093/molbev/msx149 28472384 PMC5850602

[B104] Lewsey M. Palukaitis P. Carr J. P. (2009). “Chapter 6. Plant–virus interactions: defence and counter-defence,” in Annual Plant Reviews, Molecular Aspects of plant Disease Resistance, Vol 34, ed J. Parker (Cologne: John Wiley and Sons), 134–176. 10.1111/b.9781405175326.2009.00006.x

[B105] Liang Y. Gao X. W. (2017). The cuticle protein gene MPCP4 of *Myzus persicae* (Homoptera: Aphididae) plays a critical role in cucumber mosaic virus acquisition. J. Econ. Entomol. 110, 848–853. 10.1093/jee/tox025 28334092

[B106] Liu W. Gray S. Huo Y. Li L. Wei T. Wang X. (2015). Proteomic analysis of interaction between a plant virus and its vector insect reveals new functions of hemipteran cuticular protein. Mol. Cell. Proteomics 14, 2229–2242. 10.1074/mcp.M114.046763 26091699 PMC4528249

[B107] Livak K. J. Schmittgen T. D. (2001). Analysis of relative gene expression data using real-time quantitative PCR and the 2– ΔΔCT method. Methods 25, 402–408. 10.1006/meth.2001.1262 11846609

[B108] Lorenzen J. H. Meacham T. Berger P. H. Shiel P. J. Crosslin J. M. Hamm P. B.. (2006). Whole genome characterization of Potato virus Yisolates collected in the western USA and their comparison to isolates from Europe and Canada. Arch. Virol. 151, 1055–1074. 10.1007/s00705-005-0707-6 16463126

[B109] Lukan T. Pompe-Novak M. Baebler Š.;, Tušek Žnidari?c, M. Kladnik A. Blejec A.. (2020). Spatial accumulation of salicylic acid is in effector-triggered immunity of potato against viruses regulated by RBOHD. bioRxiv. 10.1101/2020.01.06.889998

[B110] Mahajan S. Dolja V. V. Carrington J. C. (1996). Roles of the sequence encoding tobacco etch virus capsid protein in genome amplification: requirements for the translation process and a cisactive element. J. Virol. 70, 4370–4379. 10.1128/jvi.70.7.4370-4379.1996 8676460 PMC190370

[B111] Makarova S. Makhotenko A. Spechenkova N. Love A. J. Kalinina N. O. Taliansky M. (2018). Interactive responses of potato (*Solanum tuberosum* L.) plants to heat stress and infection with potato virus Y. Front. Microbiol. 9, 2582. 10.3389/fmicb.2018.02582 30425697 PMC6218853

[B112] Makhotenko A. V. Khromov A. V. Snigir E. A. Makarova S. S. Makarov V. V. Suprunova T. P.. (2019). Functional analysis of coilin in virus resistance and stress tolerance of potato Solanum tuberosum using CRISPR-Cas9 editing. Dokl. Biochem. Biophys. 484, 88–91. 10.1134/S1607672919010241 31012023

[B113] Mao Y. Sun X. Shen J. Gao F. Qiu G. Wang T.. (2019). Molecular evolutionary analysis of potato virus Y infecting potato based on the VPg gene. Front. Microbiol. 10, 1708. 10.3389/fmicb.2019.01708 31402905 PMC6676787

[B114] Martin B. Collar J. L. Tjallingii W. F. Fereres A. (1997). Intracellular ingestion and salivation by aphids may cause the acquisition and inoculation of non-persistently transmitted plant viruses. J. Gen. Virol. 78, 2701–2705. 10.1099/0022-1317-78-10-2701 9349493

[B115] Martin D. P. Murrell B. Golden M. Khoosal A. Muhire B. (2015). RDP4: Detection and analysis of recombination patterns in virus genomes. Virus Evol. 1, vev003. 10.1093/ve/vev003 27774277 PMC5014473

[B116] Mclean D. L. Kinsey M. G. (1964). A technique for electronically recording aphid feeding and salivation. Nature 202, 1358–1359. 10.1038/2021358a0

[B117] Medina Cárdenas H. C. Gutiérrez Sánchez P. A. Marín Montoya M. A. (2017). Detection and sequencing of *potato virus Y* (PVY) and *Potato leafroll virus* (PLRV) in a volunteer plant of *Solanum tuberosum* L. cv. Diacol-Capiro. Acta Agronómica 66, 625–632. 10.15446/acag.v66n4.59753

[B118] Michelmore R. W. Paran I. Kesseli R. V. (1991). Identification of markers linked to disease resistance genes by bulked segregant analysis, a rapid method to detect markers in specific genomic regions by using segregating populations. Proc. Natl. Acad. Sci. U. S. A., 88, 9828–9832. 10.1073/pnas.88.21.9828 1682921 PMC52814

[B119] Miroshnichenko D. Timerbaev V. Okuneva A. Klementyeva A. Sidorova T. Pushin A.. (2020). Enhancement of resistance to PVY in intragenic marker-free potato plants by RNAi-mediated silencing of eIF4E translation initiation factors. Plant Cell Tissue Organ Cult. 140, 691–705. 10.1007/s11240-019-01746-9

[B120] Mishra R. Verma R. K. Sharma P. Choudhary D. K. Gaur R. K. (2014). Interaction between viral proteins with the transmission of Potyvirus. Arch. Phytopathol. Plant Prot. 47, 240–253. 10.1080/03235408.2013.807659

[B121] Monsanto, (1997). “NewLeaf potatoes”, in Biotechnology Resource Guide. Naturemark potatoes. Boise. Monsanto.

[B122] Moodley V. Naidoo R. Gubba A. Mafongoya P. L. (2019). Development of potato virus Y (PVY) resistant pepper (*Capsicum annuum* L.) lines using marker-assisted selection (MAS). Physiol. Mol. Plant. Pathol. 105, 96–101. 10.1016/j.pmpp.2018.12.002

[B123] Moreno A. Bertolini E. Olmos A. Cambra M. Fereres A. (2007). Estimation of vector propensity for Lettuce mosaic virus based on viral detection in single aphids. Spanish J. Agric. Res. 5, 376–384. 10.5424/sjar/2007053-5343

[B124] Mori K. Mukojima N. Nakao T. Tamiya S. Sakamoto Y. Sohbaru N.. (2012). Germplasm release: Saikai 35, a male and female fertile breeding line carrying Solanum Phureja- derived cytoplasm and potato cyst nematode resistance (H1) and potato virus Y resistance (Ry chc) genes. Am. J. Potato. Res. 89, 63–72. 10.1007/s12230-011-9221-4

[B125] Moury B. Desbiez C. (2020). Host range evolution of potyviruses: a global phylogenetic analysis. Viruses 12, 111. 10.3390/v12010111 31963241 PMC7020010

[B126] Moury B. Simon V. (2011). dN/dS-based methods detect positive selection linked to trade-offs between different fitness traits in the coat protein of potato virus Y. Mol. Biol. Evol. 28, 2707–2717. 10.1093/molbev/msr105 21498601

[B127] Munoz F. J. Plaisted R. L. Thurston H. D. (1975). Resistance to potato virus Y in *Solanum tuberosum* spp.Andigena. Am. Potato. J. 52, 107–115. 10.1007/BF02852043

[B128] Nanayakkara U. N. Nie X. Giguère M. Zhang J. Boquel S. Pelletier Y. (2012). Aphid feeding behavior in relation to potato virus Y (PVY) acquisition. J. Econ. Entomol. 105, 1903–1908. 10.1603/EC11427 23356052

[B129] Nasr-Eldin M. Messiha N. Othman B. Megahed A. Elhalag K. (2019). Induction of potato systemic resistance against the potato virus Y (PVYNTN), using crude filtrates of *Streptomyces* spp. under greenhouse conditions. Egypt. J. Biol. Pest. Control. 29, 62. 10.1186/s41938-019-0165-1

[B130] Nasr-Eldin M. A. Othman B. A. Megahed A. A. El-Masry S. S. Faiesal A. A. (2018). Physiological, cytological and molecular analysis of PVYNTN-infected potato cultivars. Egy J. Exp. Biol. 14, 171–185. 10.5455/egyjebb.20180222091921

[B131] Nie B. Singh M. Murphy A. Sullivan A. Xie C. Nie X. (2012). Response of potato cultivars to five isolates belonging to four strains of potato virus Y. Plant. Dis. 96, 1422–1429. 10.1094/PDIS-01-12-0018-RE 30727313

[B132] Noha K. Bondok A. M. El-Dougdoug K. A. (2018). Evaluation of silver nanoparticles as antiviral agent against ToMV and PVY in tomato plants. Sciences 8, 100–111.

[B133] Novy R. G. Whitworth J. L. Stark J. C. Schneider B. L. Knowles N. R. Pavek M. J.. (2017). Payette russet: a dual-purpose potato cultivar with cold-sweetening resistance, low acrylamide formation, and resistance to late blight and potato virus Y. Am. J. Potato. Res. 94, 38–53. 10.1007/s12230-016-9546-0

[B134] O'Donnell P. J. Schmelz E. A. Moussatche P. Lund S. T. Jones J. B. Klee H. J. (2003). Susceptible to intolerance–a range of hormonal actions in a susceptible Arabidopsis pathogen response. Plant J. Cell. Mol. Biol. 33, 245–257. 10.1046/j.1365-313X.2003.01619.x 12535339

[B135] Osmani Z. Sabet M. S. Shams-Bakhsh M. Moieni A. Vahabi K. (2019). Virus-specific and common transcriptomic responses of potato (*Solanum tuberosum*) against PVY, PVA and PLRV using microarray meta-analysis. Plant. Breed 138, 216–228. 10.1111/pbr.12671

[B136] Piche L. M. Singh R. P. Nie X. Gudmestad N. C. (2004). Diversity among potato virus Y isolates obtained from potatoes grown in the United States. Phytopathology 94, 1368–1375. 10.1094/PHYTO.2004.94.12.1368 18943708

[B137] Pirone T. P. Harris K. F. (1977). Nonpersistent transmission of plant viruses by aphids. Annu. Rev. Phytopathol. 15, 55–73. 10.1146/annurev.py.15.090177.0004156444123

[B138] Polder G. Blok P. M. De Villiers H. A. Van der Wolf J. M. Kamp J. (2019). Potato virus Y detection in seed potatoes using deep learning on hyperspectral images. Front. Plant Sci., 10, 209. 10.3389/fpls.2019.00209 30881366 PMC6405642

[B139] Pollari M. De S. Wang A. Mäkinen K. (2020). The potyviral silencing suppressor HCPro recruits and employs host ARGONAUTE1 in pro-viral functions. PLoS. Pathog. 16, e1008965. 10.1371/journal.ppat.1008965 33031436 PMC7575100

[B140] Pooggin M. M. (2017). RNAi-mediated resistance to viruses: a critical assessment of methodologies. Curr. Opin. Virol. 26, 28–35. 10.1016/j.coviro.2017.07.010 28753441

[B141] Powell G. (1993). The effect of pre-acquisition starvation on aphid transmission of potyviruses during observed and electrically recorded stylet penetrations. Entomol. Exp. Appl. 66, 255–260. 10.1111/j.1570-7458.1993.tb00716.x

[B142] Powell G. (2005). Intracellular salivation is the aphid activated associated with inoculation of non-persistently transmitted viruses. J. Gen. Virol. 86, 469–472. 10.1099/vir.0.80632-0 15659767

[B143] Quenouille J. Vassilakos N. Moury B. (2013). P otato virus Y: a major crop pathogen that has provided major insights into the evolution of viral pathogenicity. Mol. Plant Pathol. 14, 439–452. 10.1111/mpp.12024 23480826 PMC6638879

[B144] Revers F. Garcia J. (2015). Molecular Biology of Potyviruses. Adv. Virus Res. 92, 101–199. 10.1016/bs.aivir.2014.11.006 25701887

[B145] Rodamilans B. Valli A. Mingot A. San León D. López-Moya J. J. García J. A. (2018). An atypical RNA silencing suppression strategy provides a snapshot of the evolution of sweet potato-infecting potyviruses. Sci. Rep. 8, 15937. 10.1038/s41598-018-34358-y 30374036 PMC6206096

[B146] Rodriguez-Rodriguez M. Chikh-Ali M. Johnson S. B. Gray S. M. Malseed N. Crump N.. (2020). The recombinant potato virus Y (PVY) strain, PVYNTN, identified in potato fields in Victoria, southeastern Australia. Plant Dis. 104, 3110–3114. 10.1094/PDIS-05-20-0961-SC 33058718

[B147] Ross B. T. Zidack N. K. Flenniken M. L. (2021). Extreme resistance to viruses in potato and soybean. Front. Plant. Sci. 12, 658981. 10.3389/fpls.2021.658981 33889169 PMC8056081

[B148] Ross H. (1958). “Inheritance of extreme resistance to potato virus Y in *Solanum stoloniferum* and its hybrids with *Solanum tuberosum*,” in Proceedings of the Third Conference Potato Virus Diseases (Lisse-Wageningen), 204–211.

[B149] Rubio L. Galipienso L. Ferriol I. (2020). Detection of plant viruses and disease management: relevance of genetic diversity and evolution. Front. Plant. Sci. 11, 1092. 10.3389/fpls.2020.01092 32765569 PMC7380168

[B150] Sanfaçon H. (2015). Plant translation factors and virus resistance. Viruses 7, 3392–3419. 10.3390/v7072778 26114476 PMC4517107

[B151] Scholthof K. B. G. Adkins S. Czosnek H. Palukaitis P. Jacquot E. Hohn T.. (2011). Top 10 plant viruses in molecular plant pathology. Mol. Plant. Pathol. 12, 938–954. 10.1111/j.1364-3703.2011.00752.x 22017770 PMC6640423

[B152] Seo J. K. Kwon S. J. Cho W. K. Choi H. S. Kim K. H. (2014). Type 2C protein phosphatase is a key regulator of antiviral extreme resistance limiting virus spread. Sci. Rep. 4, 5905. 10.1038/srep05905 25082428 PMC5379993

[B153] Setten R. L. Rossi J. J. Han S. P. (2019). The current state and future directions of RNAi-based therapeutics. Nat. Rev. Drug. Discov. 18, 421–446. 10.1038/s41573-019-0017-4 30846871

[B154] Shang F. Ding B. Y. Ye C. Yang L. Chang T. Y. Xie J.. (2020). Evaluation of a cuticle protein gene as a potential RNAi target in aphids. Pest. Manag. Sci. 76, 134–140. 10.1002/ps.5599 31461217

[B155] Shanks C. H. Chapman R. K. (1965). The effects of insecticides on the behavior of the green peach aphid and its transmission of potato virus. J. Econ. Entomol. 58, 79–83. 10.1093/jee/58.1.7929528399

[B156] Singh A. K. Chakrabarti S. K. Singh B. Sharma J. Dua V. K. (2020). Potato Science and Technology for Sub-Tropics. New Delhi: New India Publishing Agency.

[B157] Singh R. P. McLaren D. L. Nie X. Singh M. (2003). Possible escape of a recombinant isolate of potato virus Y by serological indexing and methods of its detection. Plant. Dis. 87, 679–685. 10.1094/PDIS.2003.87.6.679 30812860

[B158] Slater A. T. Schultz L. Lombardi M. Rodoni B. C. Bottcher C. Cogan N. O. I.. (2020). Screening for resistance to PVY in Australian potato germplasm. Genes 11, 429. 10.3390/genes11040429 32316258 PMC7230960

[B159] Soare E. Chiurciu I. A. (2021). Study on the Dynamics of Potato Production and Worldwide Trading During the Period 2012–2019 Changes, Romania: Scientific Papers Series Management, Economic Engineering in Agriculture and Rural Development. 21.

[B160] Solomon-Blackburn R. M. Barker H. (2001). Breeding virus resistant potatoes (*Solanum tuberosum*): a review of traditional and molecular approaches. Heredity 86, 17–35. 10.1046/j.1365-2540.2001.00799.x 11298812

[B161] Stare T. Ramšak Ž. Križnik M. Gruden K. (2019). Multiomics analysis of tolerant interaction of potato with potato virus Y. Sci. Data 6, 250. 10.1038/s41597-019-0216-1 31673114 PMC6823367

[B162] Sylvester E. S. (1980). Circulative and propagative virus transmission by aphids. Annu. Rev. Entomol. 25, 257–286. 10.1146/annurev.en.25.010180.001353

[B163] Szajko K. Strzelczyk-Zyta D. Marczewski W. (2018). Comparison of leaf proteomes of potato (*Solanum tuberosum* L.) genotypes with ER- and HR-mediated resistance to PVY infection. Eur. J. Plant Pathol. 150:375–385. 10.1007/s10658-017-1284-8

[B164] Tiwari J. K. Mandadi N. Sridhar J. Mandal V. Ghosh A. Kardile H. B.. (2021). Draft genome sequencing of the foxglove aphid (*Aulacorthum solani* Kaltenbach), a vector of potato viruses, provides insights on virulence genes. J. Asia. Pac. Entomol. 24, 93–102. 10.1016/j.aspen.2021.03.010

[B165] Tjallingii W. F. (1978). Electronic recording of penetration behaviour by aphids. Entomol. Exp. Appl. 24, 721–730. 10.1111/j.1570-7458.1978.tb02836.x

[B166] Tjallingii W. F. (1988). “Electrical recording of stylet penetration activities,” in Aphids: Their Biology, Natural Enemies and Control, 2b, eds A. K. Minks and P. Harrewijn (Amsterdam: Elsevier), 95–108.

[B167] Torrance L. Cowan G. H. McLean K. MacFarlane S. Al-Abedy A. N. Armstrong M.. (2020). Natural resistance to potato virus Y in *Solanum tuberosum* Group Phureja. TAG. Theor. Appl. Genet. 133, 967–980. 10.1007/s00122-019-03521-y 31950199 PMC7021755

[B168] Tyagi S. Kesiraju K. Saakre M. Rathinam M. Raman V. Pattanayak D.. (2020). Genome editing for resistance to insect pests: an emerging tool for crop improvement. ACS Omega 5, 20674–20683. 10.1021/acsomega.0c01435 32875201 PMC7450494

[B169] Uzest M. Gargani D. Drucker M. Hébrard E. Garzo E. Candresse T.. (2007). A protein key to plant virus transmission at the tip of the insect vector stylet. Proc. Natl. Acad. Sci. U. S. A. 104, 17959–17964. 10.1073/pnas.0706608104 17962414 PMC2084279

[B170] Valkonen J. P. T. (1994). Natural genes and mechanisms for resistance to viruses in cultivated and wild potato species (*Solanum* spp.). Plant Breed 112, 1–16. 10.1111/j.1439-0523.1994.tb01270.x

[B171] Valkonen J. P. T. (2007). “Viruses: economical losses and biotechnological potential,” in Potato Biology and Biotechnology: Advances and Perspectives (Helsinki: Elsevier), 619–641. 10.1016/B978-044451018-1/50070-1

[B172] Valkonen J. P. T. (2015). Elucidation of virus–host interactions to enhance resistance breeding for control of virus diseases in potato. Breed. Sci. 65, 69–76. 10.1270/jsbbs.65.69 25931981 PMC4374565

[B173] Valkonen J. P. T. Palohuhta J. P. (1996). Resistance to potato virus A and potato virus Y in potato cultivars grown in Finland. AF Sci. 5, 57–62. 10.23986/afsci.72730

[B174] Valli A. A. Gallo A. Rodamilans B. López-Moya J. J. García J. A. (2018). The HCPro from the Potyviridae family: An enviable multitasking helper component that every virus would like to have. Mol. Plant Pathol. 19, 744–763. 10.1111/mpp.12553 28371183 PMC6638112

[B175] Van Munster M. (2020). Impact of abiotic stresses on plant virus transmission by aphids. Viruses 12, 216. 10.3390/v12020216 32075208 PMC7077179

[B176] Verbeek M. Piron P. G. M. Dullemans A. M. Cuperus C. Van Der Vlugt R. A. A. (2010). Determination of aphid transmission efficiencies for N,N-TN and Wilga strains of potato virus Y. Ann Appl. An. Appl. Biol. 156, 39–49. 10.1111/j.1744-7348.2009.00359.x

[B177] Verchot J. Koonin E. V. Carrington J. C. (1991). The 35-kDa protein from the N-terminus of the potyviralpolyprotein functions as a third virus-encoded proteinase. Virology 185, 527–535. 10.1016/0042-6822(91)90522-D 1962435

[B178] Vidal S. Cabrera H. Andersson R. A. Fredriksson A. Valkonen, J. P. (2002) Potato gene Y-1 is an N gene homolog that confers cell death upon infection with potato virus Y. Mol. Plant Microbe Interact. 15, 717–727. 10.1094/MPMI.2002.15.7.717 12118888

[B179] Wang B. Ma Y. Zhang Z. Wu Z. Wu Y. Wang Q.. (2011). Potato viruses in China. Crop. Prot. 30, 1117–1123. 10.1016/j.cropro.2011.04.001

[B180] Wang R. Y. Ammuar E. D. Thornbury D. W. Lopez-Moya J. J. Pirone T. P. (1996). Loss of Potyvirus transmissibility and helper-component activity correlate with non-retention of virions in aphid stylets. J. Gen. Virol. 77, 861–867. 10.1099/0022-1317-77-5-861 8609482

[B181] Warren M. Kruger K. Schoeman A. (2005). *Potato virus Y (PVY) and Potato Leafroll Virus (PLRV)*. A South African Perspective. Pretoria: University of Pretoria.

[B182] Webster C. G. Pichon E. Van Munster M. Monsion B. Deshoux M. Gargani D.. (2018). Identification of plant virus receptor candidates in the stylets of their aphid vectors. J. Virol. 92, e00432-18. 10.1128/JVI.00432-18 29769332 PMC6026765

[B183] Webster C. G. Thillier M. Pirolles E. Cayrol B. Blanc S. Uzest M. (2017). Proteomic composition of the acrostyle: novel approaches to identify cuticular proteins involved in virus-insect interactions. Insect Sci. 24, 990–1002. 10.1111/1744-7917.12469 28421675 PMC5724696

[B184] Were H. K. Kabira J. N. Kinyua Z. M. Olubayo F. M. Karinga J. K. Aura J.. (2013). Occurrence and distribution of potato pests and diseases in Kenya. Potato. Res. 56, 325–342. 10.1007/s11540-013-9246-9

[B185] Wesley P. F. M. Luciano V. P. (2019). RNA interference: a promising molecular tool for insect-pest control. Mod. Concep. Dev. Agron. 5. 10.31031/MCDA.2019.05.000614

[B186] Whitham S. A. Yang C. Goodin M. M. (2006). Global impact: elucidating plant responses to viral infection. Mol. Plant. Microbe. Interact. 19, 1207–1215. 10.1094/MPMI-19-1207 17073303

[B187] Wickham H. Chang W. Wickham M. H. (2013). Package'ggplot2. New York, NY: Verlag.

[B188] Witek K. Jupe F. Witek A. I. Baker D. Clark M. D. Jones J. D. (2016). Accelerated cloning of a potato late blight–resistance gene using RenSeq and SMRT sequencing. Nat. Biotechnol. 34, 656–660. 10.1038/nbt.3540 27111721

[B189] Wolf Y. I. Kazlauskas D. Iranzo J. Lucía-Sanz A. Kuhn J. H. Krupovic M.. (2018). Origins and evolution of the global RNA virome. mBio. 9, e02329–e02318. 10.1128/mBio.02329-18 30482837 PMC6282212

[B190] Wróbel S. (2009). The retention of PVY in the stylet of *Myzus persicae* Sulz. after the application of mineral oil on potato plants. Plant. Breed. Seed Sci. 60, 3–12. 10.2478/v10129-010-0001-y

[B191] Wylie S. J. Adams M. Chalam C. Kreuze J. López-Moya J. J. Ohshima K.. (2017). ICTV virus taxonomy profile: potyviridae. J. Gen. Virol. 98, 352–354. 10.1099/jgv.0.000740 28366187 PMC5797945

[B192] Yin Z. Xie F. Michalak K. Pawełkowicz M. Zhang B. Murawska Z.. (2017). Potato cultivar Etola exhibits hypersensitive resistance to PVY ^NTN^ and partial resistance to PVY^Z − NTN^ and PVY^N − Wi^ strains and strain-specific alterations of certain host miRNAs might correlate with symptom severity. Plant. Pathol. 66, 539–550. 10.1111/ppa.12599

[B193] Yu N. Christiaens O. Liu J. Niu J. Cappelle K. Caccia S.. (2013). Delivery of dsRNA for RNAi in insects: an overview and future directions. Insect. Sci. 20, 4–14. 10.1111/j.1744-7917.2012.01534.x 23955821

[B194] Zhan X. Zhang F. Zhong Z. Chen R. Wang Y. Chang L.. (2019). Generation of virus resistant potato plants by RNA genome targeting. Plant Biotechnol J. 17, 1814–1822. 10.1111/pbi.13102 30803101 PMC6686122

[B195] Zhang J. Nie X. Nanayakkara U. Boquel S. Giguère M. A. Pelletier Y. (2013). Detection of potato virus Y from the stylets of a single aphid by one-step reverse transcription polymerase chain reaction. Entomol. Exp. Appl. 147, 93–97. 10.1111/eea.12044

[B196] Zhu K. Y. (2013). RNA interference: a powerful tool in entomological research and a novel approach for insect pest management. Insect. Sci. 20, 1–3. 10.1111/1744-7917.12006 23955820

